# Exosomal POSTN from cancer-associated fibroblasts drives progression of microinvasive lung adenocarcinoma: insights from single-cell and tissue exosome sequencing analysis

**DOI:** 10.3389/fimmu.2026.1767771

**Published:** 2026-05-19

**Authors:** Jie Chen, Chenkang Zhou, Yixin Chen, Kate Huang, Yumin Wang, Junjun Wang

**Affiliations:** 1 Department of Intensive Care Unit, The First Affiliated Hospital of Wenzhou Medical University, Wenzhou, China; 2 Department of Laboratory Medicine, The First Affiliated Hospital of Wenzhou Medical University, Wenzhou, China; 3 Department of Pathology, The First Affiliated Hospital of Wenzhou Medical University, Wenzhou, China

**Keywords:** cancer-associated fibroblasts, exosomes, lung adenocarcinoma, microinvasive adenocarcinoma, postn

## Abstract

**Background:**

Microinvasive adenocarcinoma (MIA) represents an early stage of lung adenocarcinoma (LUAD), yet how the tumor microenvironment (TME) and cancer-associated fibroblast (CAF)-derived exosomes contribute to its progression remains unclear. We aimed to define the cellular ecosystem of MIA and to clarify the role of periostin (POSTN) and POSTN^+^ CAF-derived exosomes in early LUAD progression.

**Methods:**

Single-cell RNA sequencing (scRNA-seq) and tissue-derived exosomal RNA sequencing were performed on four primary MIA lesions and matched adjacent lung tissues. Integrated analyses of scRNA-seq data, exosomal transcriptomes, the TCGA-LUAD cohort, and an independent LUAD tissue/serum cohort were used to characterize POSTN expression and to evaluate its prognostic and diagnostic relevance. Primary MIA-associated POSTN^+^ and POSTN^-^ CAFs were isolated for exosome preparation, followed by co-culture experiments with LUAD cell lines and xenograft assays.

**Results:**

scRNA-seq identified a malignant Cancer–alveolar type II (Cancer-AT2) epithelial subset and multiple CAF subsets. Among these, POSTN^+^ CAFs were enriched in MIA tissues and showed enhanced crosstalk with Cancer-AT2 cells through extracellular matrix (ECM)-related ligand–receptor interactions. Tissue-derived exosomes contained 588 differentially expressed mRNAs, among which POSTN was markedly upregulated and showed the strongest association with fibroblast-related signatures. POSTN was predominantly expressed in fibroblasts across independent non-small cell lung cancer datasets and was elevated in LUAD tissues, tissue-derived exosomes, and serum exosomes, correlating with advanced stage, lymph node metastasis, and poor survival. Functionally, POSTN^+^ CAF-derived exosomes promoted LUAD cell proliferation, migration, invasion, colony formation, and xenograft growth.

**Conclusion:**

Exosomal POSTN derived from POSTN^+^ CAFs may represent an important stromal mediator of MIA/LUAD progression and a potential diagnostic and prognostic biomarker in early-stage LUAD.

## 1 Introduction

Lung cancer remains a formidable global health challenge, with incidence and mortality rates in China accounting for 17.9% and 23.8%, respectively, of all malignant tumors in 2020 (1). Among its subtypes, lung adenocarcinoma (LUAD) has shown a steadily rising incidence worldwide and exhibits a higher prevalence in females (2). A critical concern in its management is that approximately 75% of patients are diagnosed at an advanced stage, thereby missing the window for curative surgical intervention (2). Robust evidence underscores the life-saving value of early detection: while the 5-year survival for Stage IIIA–IV LUAD patients is only 10–36%, it rises dramatically to 77–92% for Stage I patients. Notably, both adenocarcinoma *in situ* (AIS) and microinvasive adenocarcinoma (MIA) achieve nearly 100% 5-year disease-specific survival after complete resection, highlighting the crucial importance of early diagnosis (3, 4). It is precisely at the transitional stage of MIA, where malignant cells have begun to breach the basement membrane but have not yet gained full invasive potential, that the molecular drivers of progression become activated (5, 6). Therefore, elucidating the mechanisms underlying MIA progression represents a pivotal opportunity to alter the natural history of LUAD, potentially unveiling novel biomarkers for monitoring early disease evolution and therapeutic targets for preventing advancement to invasive carcinoma.

Exosomes play a crucial role in intercellular communication by transferring bioactive molecules—including DNA, various RNA species, and proteins—between cells (7). They have been extensively implicated in key tumorigenic processes such as metastasis, invasion, and angiogenesis (8). By mediating dialogue between cancer cells and the tumor microenvironment (TME), exosomes promote epithelial-mesenchymal transition (EMT), angiogenesis, and immune evasion, thereby accelerating cancer progression (9). While most previous studies have relied on exosomes isolated from peripheral blood or cell culture systems, growing evidence highlights the unique value of exosomes derived directly from tumor tissues. Such tissue-derived exosomes more faithfully mirror the complex molecular landscape of the native TME, which includes heterogeneous signals from tumor cells, stromal components, and immune cells (10, 11). In contrast, conventional cell culture models lack the intricate multicellular crosstalk of actual tumor tissue, limiting their ability to fully recapitulate TME dynamics (12, 13). Despite their potential, the role of tissue-derived exosomes, particularly in early-stage lesions such as MIA, remains underexplored. A deeper understanding of these exosomes could provide critical insights into the initial molecular events driving LUAD progression.

Single-cell RNA sequencing (scRNA-seq) has transformed cancer research by resolving gene expression at single-cell resolution, enabling detailed dissection of intratumoral heterogeneity and the TME (14, 15). In LUAD, scRNA-seq studies have revealed diverse malignant epithelial states and distinct stromal and immune cell subsets, linking specific TME patterns to radiologic phenotypes and patient outcomes (16). In parallel, exosomal RNA profiling has shown that tumor-derived exosomal mRNAs and non-coding RNAs actively drive lung cancer growth, metastasis, therapy resistance, and immune evasion, while also offering promise for early diagnosis and prognostic stratification (17). Recent work has begun to couple exosome-related gene programs with single-cell–defined cell populations in the TME, highlighting the value of integrating cellular-resolution atlases with exosome-centered analyses to decode tumor–stroma crosstalk in LUAD (18). Despite these advances, critical gaps remain, particularly in early-stage lesions such as MIA, which represent a pivotal transition between adenocarcinoma *in situ* and fully invasive disease. Here, based on the integrated analysis of single-cell and tissue exosome sequencing, this study aims to systematically investigate the cellular dynamics and intercellular communication mechanisms within the MIA microenvironment. We seek to identify key stromal cell subpopulations that drive early tumor progression through exosome-mediated signaling and to delineate their functional roles in promoting malignant transformation. The research employs a multi-level validation strategy to establish the biological significance of the identified cellular crosstalk in MIA development. By elucidating the complex cellular interactions at this critical transitional stage, our findings provide novel insights into the early pathogenesis of LUAD and offer potential biomarkers for early detection and therapeutic targets for preventing disease progression. This work not only advances our fundamental understanding of MIA biology but also holds significant promise for improving clinical outcomes through early intervention strategies.

## 2 Materials and methods

### 2.1 Sample collection

**Figure 1:**
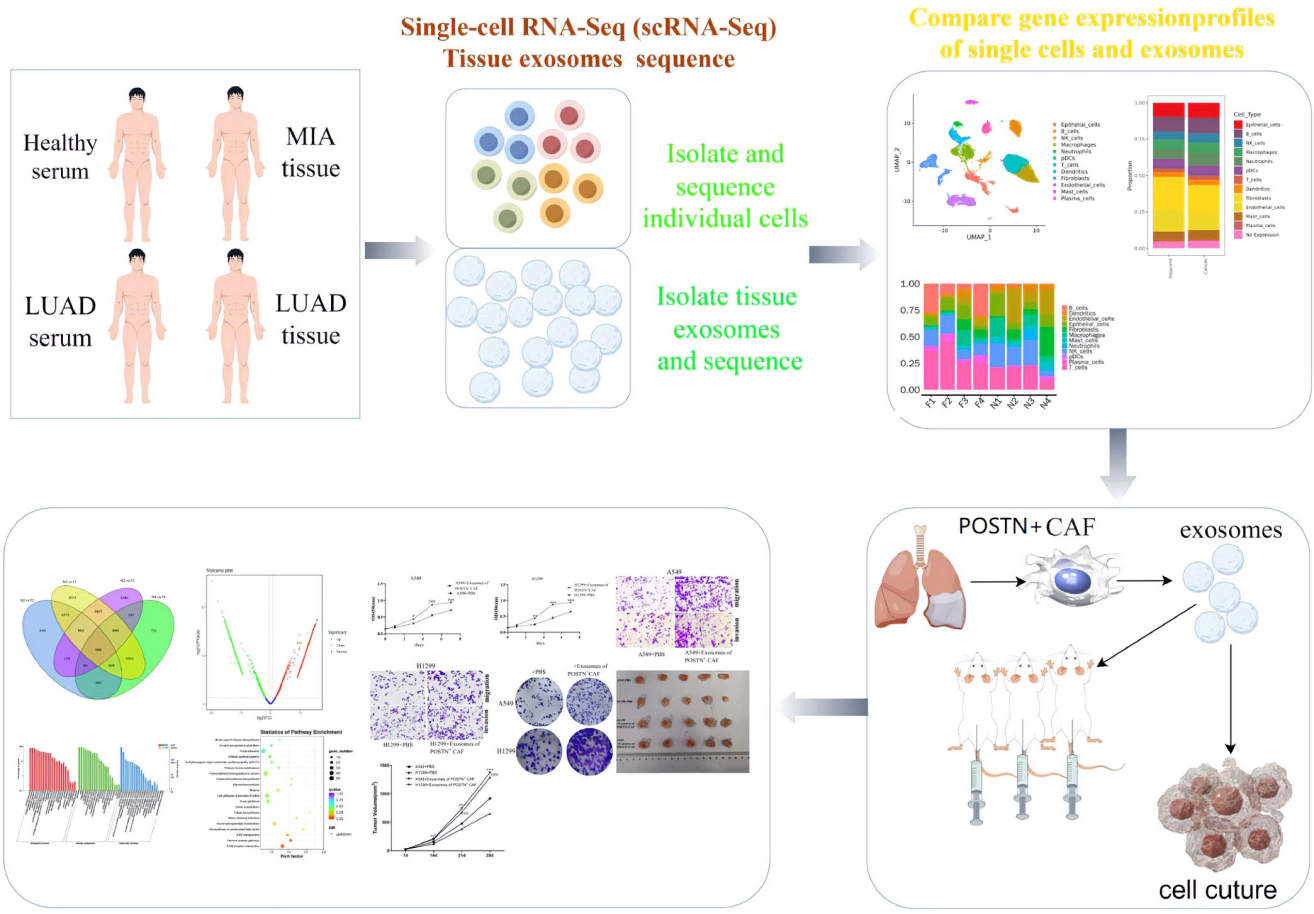
Flow chart of this study.

The overall experimental design and workflow are presented in **Figure 1**. Cancer tissues and matched adjacent normal tissues were collected from four patients with MIA who underwent surgery at the First Affiliated Hospital of Wenzhou Medical University between April 5 and April 10, 2022 (**Supplementary Table 1**). All samples were transported to Echo Biotech Company and processed within 8 hours of collection. All patients were diagnosed with stage I LUAD. The inclusion criteria were as follows: (I) histopathological confirmation of diagnosis; (II) age between 18 and 80 years; (III) no prior palliative surgery, neoadjuvant chemotherapy, or radiotherapy; (IV) absence of significant organ dysfunction unrelated to the malignant disease; (V) no history of major neurological or psychiatric conditions; and (VI) no severe renal impairment, cardiovascular disease, cerebrovascular disease, hematological disorders, endocrine abnormalities, metabolic diseases, or mental illness.

Additionally, a total of 50 LUAD tissue samples and matched adjacent normal tissues were collected and pathologically confirmed. This cohort included 4 cases at stage I, 11 at stage II, 17 at stage III, and 15 at stage IV. Peripheral blood samples were also obtained from 30 treatment-naive LUAD patients and 20 healthy donors. Plasma was separated from blood cells by centrifugation at 1000 × g for 10 minutes at 4 °C. The harvested plasma was aliquoted and stored at –80 °C until further analysis.

### 2.2 Extraction and identification of tissue exosomes

Extracellular vesicles (EVs) were isolated from tissue specimens using a modified protocol based on the method of Vella et al. (19). Tissue dissociation was performed using the Human Tumor Dissociation Kit (Miltenyi Biotec, cat. no. 130-095-929) according to the manufacturer’s instructions. Exosome preparations were further enriched by ultracentrifugation, and total protein concentration was measured using a bicinchoninic acid assay (20). The morphology of the isolated vesicles was examined by transmission electron microscopy, and exosomal identity was verified by western blot detection of the canonical markers TSG101, CD63, and CD81.

### 2.3 Library preparation for scRNA-seq analysis

Single-cell transcriptome libraries were prepared using the Chromium Single Cell 3′ Library and Gel Bead Kit v3.1 (10x Genomics) according to the manufacturer’s instructions. Briefly, single-cell suspensions were loaded onto the Chromium Controller to generate gel bead-in-emulsions (GEMs), in which individual cells were partitioned together with barcoded gel beads. Reverse transcription was performed within GEMs to produce barcoded full-length cDNA, which was then recovered and amplified by PCR. Amplified cDNA was subsequently subjected to enzymatic fragmentation to yield fragments of approximately 200–300 bp, followed by end repair and A-tailing. After size selection, P7 adapters were ligated, and sample indices were introduced by PCR amplification. A final size-selection step was performed to obtain the sequencing-ready libraries. Library quality and fragment size distribution were assessed before sequencing. The libraries were then sequenced on an Illumina NovaSeq 6000 platform using paired-end 150-bp reads.

### 2.4 Single-cell sequencing data processing and bioinformatics analysis

Raw sequencing reads were first subjected to quality assessment using FastQC (v0.11.2). Downstream processing was performed with the Cell Ranger pipeline (v4.0.0), including sample demultiplexing, barcode processing, alignment to the human reference genome (GRCh38), transcript counting, and generation of unique molecular identifier (UMI)-based gene expression matrices. Subsequent analyses were conducted in R using the Seurat package (v4.0.3). Cells were retained if they expressed between 200 and 7, 000 genes and had a mitochondrial gene proportion of no more than 25%. After quality filtering, the data were normalized using the NormalizeData function with a scale factor of 10, 000, and the top 2, 000 highly variable genes were identified using FindVariableFeatures. Principal component analysis was performed on the variable genes, and batch effects across samples were corrected in PCA space using Harmony (v1.0). The batch-corrected data were then used for downstream dimensionality reduction, clustering, and visualization.

A total of 21 cell subpopulations were identified. Immune cell populations were annotated using the SingleR package with the Blueprint_Encode and HPCA reference datasets, whereas stromal and epithelial populations were assigned on the basis of established canonical marker genes. Malignant epithelial cells were further distinguished by copy number variation analysis using inferCNV. Cell type-specific marker genes were identified with Seurat’s FindAllMarkers function using the thresholds min.pct = 0.25 and logfc.threshold = 0.25. Differential expression analysis between tumor and matched adjacent tissues was performed using FindMarkers with the criteria min.pct = 0.1, adjusted *P* < 0.01, and |log2FC| > 0.5. Cell–cell communication analysis was carried out using CellChat (v1.5.0). Based on the CellChatDB ligand–receptor database, communication probabilities between cell types were inferred using the computeCommunProb function, and differences in interaction number and strength between conditions were compared using compareInteractions.

### 2.5 Tissue exosome sequencing

Total RNA was extracted from tissue-derived exosomes using the Exosome RNA Purification Kit (Simgen, cat. no. 5202050) according to the manufacturer’s instructions. RNA concentration and purity were assessed using the RNA Nano 6000 Assay Kit on an Agilent 2100 Bioanalyzer system (Agilent Technologies, CA, USA). Only RNA samples that met the quality requirements for library preparation were used for subsequent sequencing. Sequencing libraries were constructed from 4.5 ng of total RNA per sample using the SMARTer Stranded Total RNA-Seq Kit v2 (Takara Bio USA, Inc.) following the manufacturer’s protocol. Briefly, exosomal RNA was reverse-transcribed and converted into strand-specific cDNA, after which library fragments were generated and amplified. Unique index codes were added to each sample to enable multiplexed sequencing. The quality and fragment size distribution of the libraries were evaluated using the Agilent 2100 Bioanalyzer, and library quantification was further verified by qPCR. Indexed libraries were pooled and clustered on a cBot Cluster Generation System using the TruSeq PE Cluster Kit v3-cBot-HS (Illumina, San Diego, CA, USA). After cluster generation, the libraries were sequenced on an Illumina NovaSeq 6000 platform to generate paired-end 150-bp reads at EchoBiotech Co., Ltd. (Beijing, China). The resulting sequencing data were used for downstream transcriptomic profiling and differential expression analysis of tissue-derived exosomal RNAs.

### 2.6 Cell culture and processing

Two LUAD cell lines, A549 (ATCC^®^ CRM-CCL-185™) and H1299 (ATCC^®^ CRL-5803™), were obtained from the Cell Bank of the Chinese Academy of Sciences (Shanghai, China). Both cell lines were cultured in RPMI-1640 medium (Gibco, Waltham, MA, USA) supplemented with 10% fetal bovine serum (FBS, Gibco) and maintained at 37 °C in a humidified incubator with 5% CO_2_.

Primary CAFs were isolated from cancer tissues of MIA patients using differential adhesion after digestion with a trypsin–collagenase mixture. Isolated CAFs were identified by vimentin immunofluorescence and maintained in complete RPMI-1640 medium containing FBS, growth supplements, penicillin, and streptomycin. POSTN^+^ CAFs and POSTN^-^ CAFs were isolated using an immunomagnetic bead separation system (Thermo Fisher Scientific, MD, USA). In this procedure, cells expressing POSTN surface antigen were bound to magnetic beads conjugated with a POSTN antibody and retained in a magnetic field, while POSTN^-^ cells were removed during washing. Exosomes derived from POSTN^+^ CAFs were isolated using an analogous immunomagnetic procedure.

### 2.7 Cell proliferation assay

Cell proliferation was assessed using the Cell Counting Kit-8 (CCK-8; DOJINDO, Japan). Cells were seeded in 96-well plates at a density of 4 × 10³ cells per well and treated with either exosomes or phosphate-buffered saline (PBS) as a control. The CCK-8 assay was performed daily for four consecutive days. Briefly, 10 μL of CCK-8 solution was added to each well, followed by incubation at 37 °C for 1 hour. Absorbance was then measured at 450 nm using a SpectraMax microplate reader (Molecular Devices, USA).

### 2.8 Colony formation assay

Cells were seeded in 6-well plates at a density of 5 × 10² cells per well and treated with either exosomes or PBS as a control. Cells were cultured in 2 mL of medium for 14 days, with the medium replaced every four days. After the incubation period, colonies were photographed and counted. Colonies were then fixed with 4% paraformaldehyde for 30 minutes and stained with 0.2% crystal violet (Beyotime, Shanghai, China) for 15 minutes.

### 2.9 Transwell assay

Cell migration and invasion were assessed using 24-well Transwell plates with 8 μm pore filters (Corning, Tewksbury, MA, USA). For both assays, LUAD cells (4 × 10^4^ cells in 100 μL) treated with exosomes or PBS were seeded in serum-free medium in the upper chamber, while the lower chamber contained medium supplemented with 20% FBS as a chemoattractant. For the invasion assay, the upper chamber was pre-coated with Corning Matrigel. After 48 hours of incubation, cells that migrated or invaded through the membrane were fixed with 4% paraformaldehyde and stained with 0.2% crystal violet.

### 2.10 RNA extraction and RT-qPCR

Total RNA was extracted from tissues, cultured cells, and exosomes using TRIzol reagent (Invitrogen, CA, USA) according to the manufacturer’s instructions. cDNA was synthesized from 0.5 μg of total RNA using a cDNA synthesis kit (Takara, Japan). Quantitative real-time PCR was performed on a QuantStudio 5 Real-Time PCR System (Applied Biosystems, Foster City, CA, USA) using SYBR Green reagents (Takara). Gene expression levels were normalized to β-actin as the endogenous control, and relative mRNA expression was calculated using the 2^−ΔΔCt^ method. Amplification specificity was assessed by melting curve analysis. Gene-specific primers were synthesized by Sangon Biotech (Shanghai, China) with the following sequences: β-actin-F: 5′−GACGGCTACCCGATCTCGGCAT−3′, β-actin-R: 5′−ACGGCTTTCCAGCGCATCCGCA−3′, POSTN-F: 5′−CTGCTTCAGGGAGACA CACC−3′, POSTN-R: 5′−ACCACAGGAGGCTAACTCCA−3′.

### 2.11 Nude mice tumor experiment

BALB/c nude mice (female, 4–6 weeks old, 18–22 g) were maintained under specific pathogen-free (SPF) conditions. For xenograft establishment, A549 or H1299 cells (5 × 10^6^ cells per mouse) were suspended in 200 μL phosphate-buffered saline (PBS) and injected subcutaneously into the right flank of each mouse. Tumor growth was allowed to proceed for approximately 2 weeks until the xenograft volume reached about 100 mm³. At that time, 10 μg of exosomes isolated from lung adenocarcinoma-derived CAFs were injected directly into the tumor center, while mice in the control group received an equal volume of PBS. Each group contained five mice. Tumor growth was monitored over time, and tumor size was measured with calipers. Tumor volume was calculated using the formula: V = ½ × (long diameter × short diameter²). Tumor growth curves were generated on the basis of serial volume measurements. At the end of the experiment, mice were euthanized and xenograft tumors were surgically excised for gross assessment and light microscopic examination.

### 2.12 Statistical analysis

All statistical analyses were performed using GraphPad Prism 8 and R software (version 4.0.3). Continuous variables were analyzed according to the distribution characteristics of the data. For comparisons between two independent groups, a two-tailed unpaired t-test was used for normally distributed data, whereas the Wilcoxon rank-sum test was applied for data that did not meet the assumption of normality. For paired comparisons, a paired t-test or Wilcoxon signed-rank test was used as appropriate. For comparisons among more than two groups, one-way analysis of variance (ANOVA) or the Kruskal–Wallis test was used according to the data distribution. Survival analysis was performed using the survival and survminer R packages. Kaplan–Meier survival curves were generated to evaluate overall survival, and differences between groups were assessed using the log-rank test. Diagnostic performance was evaluated by receiver operating characteristic (ROC) curve analysis, and the area under the curve (AUC) was calculated to assess the discriminatory ability of candidate genes. All tests were two-sided unless otherwise specified. A *P* value of less than 0.05 was considered statistically significant.

## 3 Results

### 3.1 Single-cell landscape and immune remodeling in MIA and adjacent lung tissues

**Figure 2:**
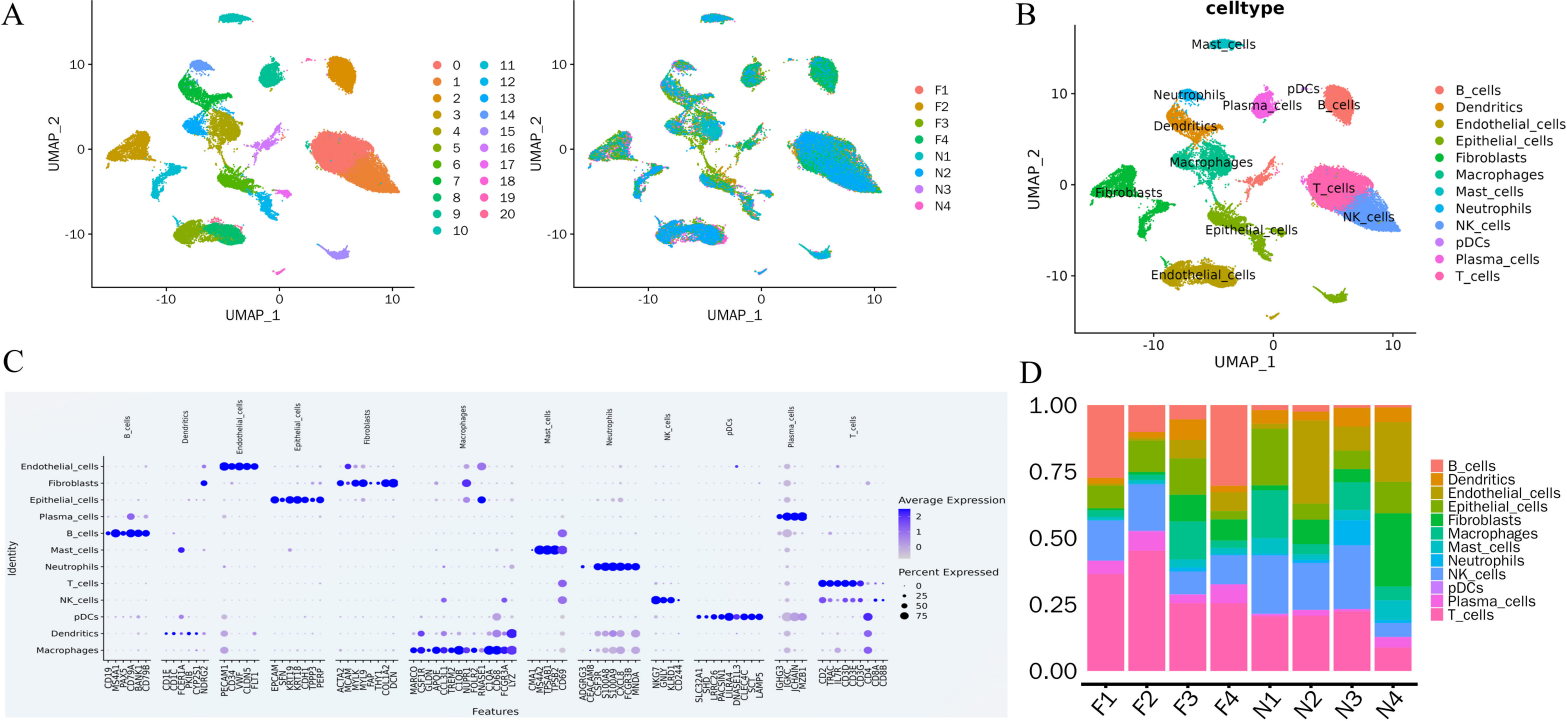
Single-cell transcriptomic landscape of MIA and matched adjacent lung tissues. **(A)** UMAP visualization of all cells colored by unsupervised clusters and by sample origin. **(B)** UMAP plot showing the annotation of major cell types, including B cells, dendritic cells, endothelial cells, epithelial cells, fibroblasts, macrophages, mast cells, neutrophils, NK cells, plasmacytoid dendritic cells (pDCs), plasma cells, and T cells. **(C)** Dot plot showing the expression of canonical marker genes used for cell-type annotation. **(D)** Stacked bar plot showing the relative proportions of major cell populations across individual samples.

After quality control, a total of 40, 413 high-quality cells from four primary MIA lesions and their matched adjacent tissues were retained for analysis. These cells were grouped into 20 transcriptionally distinct clusters (**Figure 2A**). Based on canonical marker expression, the clusters were further consolidated into 12 major cell types, including epithelial cells, natural killer (NK) cells, B cells, macrophages, neutrophils, plasmacytoid dendritic cells (pDCs), T cells, dendritic cells, fibroblasts, endothelial cells, mast cells, and plasma cells (**Figures 2B, C**). Comparison of cell-type composition between tumor and adjacent tissues revealed a pronounced remodeling of the immune microenvironment in MIA. Tumor tissues contained substantially higher proportions of B lymphocytes, T lymphocytes, and plasma cells, whereas the proportion of NK cells was markedly reduced relative to adjacent normal tissues (**Figure 2D**).

### 3.2 Global remodeling of intercellular communication networks and signaling pathways in MIA

CellChat analysis revealed substantial remodeling of the intercellular communication landscape between MIA lesions and matched adjacent lung tissues. In adjacent tissues, the strongest overall interactions were mainly concentrated among NK cells, endothelial cells, and T cells, with dense local signaling also observed among macrophages, pDCs, and dendritic cells (**Supplementary Figure 1A**). By contrast, MIA tissues displayed a more broadly reorganized network in which T cells emerged as a central communication hub, accompanied by increased connectivity of B cells, epithelial cells, fibroblasts, and endothelial cells (**Supplementary Figure 1B**). Additionally, stromal and epithelial participation in tumor tissues, including stronger fibroblast-derived and endothelial-derived signaling, although epithelial-to-macrophage communication remained more prominent than fibroblast-to-macrophage signaling (**Supplementary Figures 1C, D**).

Endothelial cell signaling also shifted between tissue types: in adjacent tissues, endothelial cells showed lower signal intensity than macrophages (**Supplementary Figure 2A**), whereas in tumor tissues their signaling became markedly stronger and exceeded that of macrophages (**Supplementary Figure 2B**). Representative ligand–receptor analysis further supported this shift, showing condition-specific interaction repertoires and stronger ECM-associated communication in tumor tissues than in adjacent tissues (**Supplementary Figure 2C**). Together, these findings indicate that early MIA is associated with marked rewiring of immune–stromal communication networks.

### 3.3 Reorganization of outgoing and incoming communication patterns in MIA

Comparative analysis of cell–cell communication between MIA lesions and matched adjacent tissues revealed marked differences in signaling pathway usage. Tumor tissues lost several pathways that were prominent in adjacent tissues while acquiring additional pathways that were not evident in the normal microenvironment (**Supplementary Figures 3A, B**). Although the overall patterns of outgoing signaling across major cell populations were broadly similar between the two conditions, incoming signaling profiles differed substantially (**Supplementary Figures 3C, D**). In adjacent tissues, endothelial cells, fibroblasts, and epithelial cells displayed distinct incoming signaling patterns. In contrast, these three cell types converged toward a nearly identical incoming pattern in tumor tissues, suggesting a more coordinated stromal and epithelial response to microenvironmental cues. Moreover, whereas immune cells in adjacent tissues largely shared a common incoming signaling profile, those in tumor tissues were separated into two distinct reception patterns, designated pattern 1 and pattern 3 (**Supplementary Figures 3E, F**). Collectively, the observed organization of incoming and outgoing signaling patterns may be associated with the increased abundance and altered organization of immune cells within the tumor microenvironment, as compared with adjacent normal tissue.

### 3.4 Identification of Cancer-AT2 as the predominant malignant epithelial subpopulation in MIA

**Figure 3:**
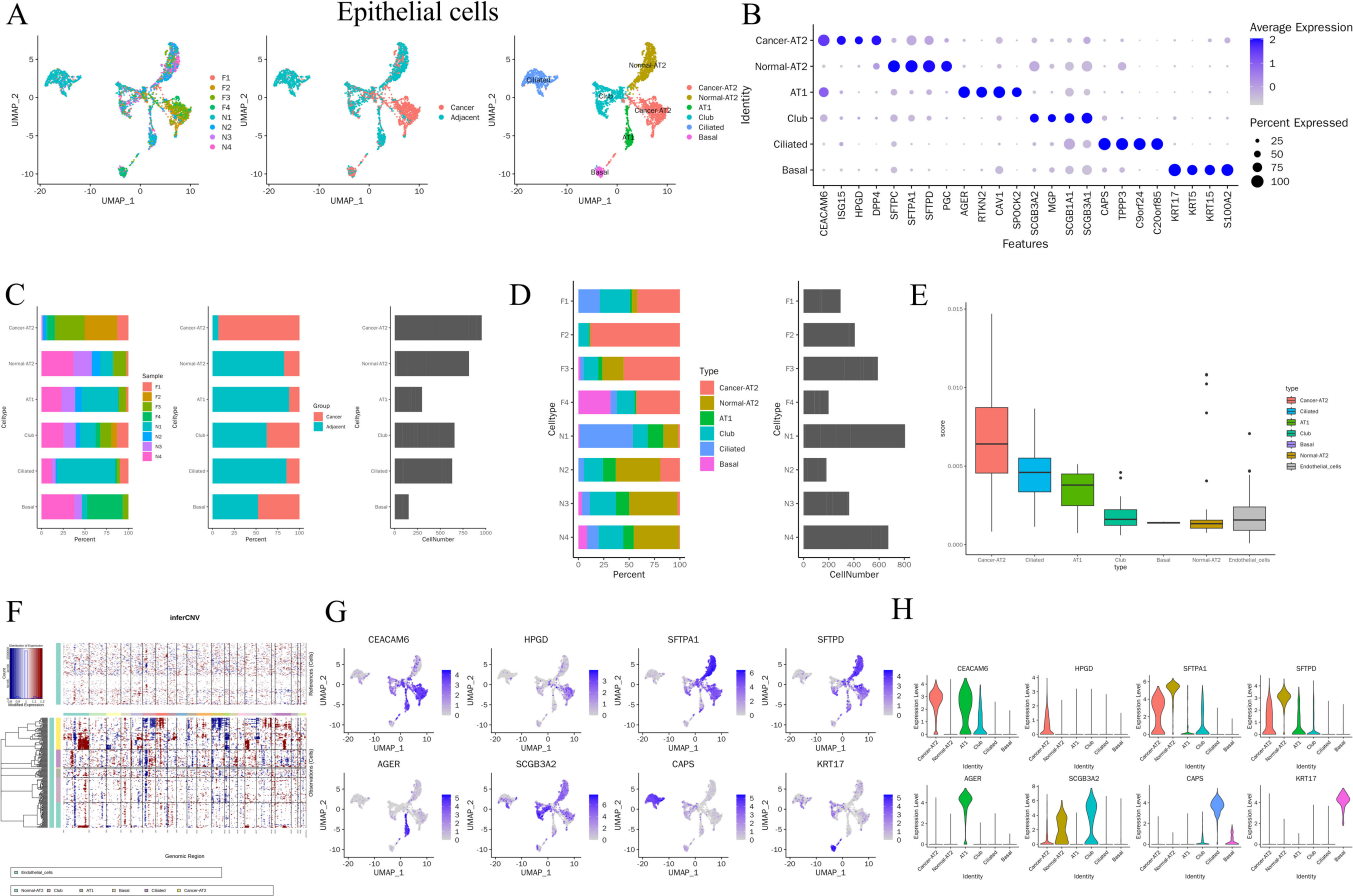
Epithelial cell heterogeneity and identification of the malignant Cancer-AT2 subpopulation in MIA. **(A)** Epithelial cells were divided into 6 subsets, including Cancer-AT2, Normal-AT2, AT1, Club, Ciliated, Basal. **(B)** Markers for epithelial cell subsets. **(C, D)** Summary of epithelial cell subset proportions in carcinoma and adjacent tissues. **(E, F)** CNV analysis shows that the Cancer-AT2 cell population has the highest malignancy level among these subsets. **(G)** Feature plots of representative epithelial marker genes. **(H)** Violin plots showing subtype-specific expression of representative marker genes.

To delineate the epithelial landscape of MIA and identify the predominant malignant epithelial subpopulation, we performed a refined analysis of epithelial cells within the tumor microenvironment. Epithelial cells were classified into six subpopulations, including Cancer-AT2, Normal-AT2, AT1, Club, Ciliated, and Basal cells (**Figures 3A, B**). Among these, Cancer-AT2, Normal-AT2, and Club cells accounted for the largest fractions (**Figure 3C**). Notably, the proportion of Cancer-AT2 cells was markedly higher in tumor tissues than in adjacent tissues (93.41% vs. 6.59%; **Figure 3D**), indicating an expansion of malignant epithelial cells within MIA lesions. Cancer-AT2 also represented the largest epithelial subpopulation overall, and showed the highest malignancy score among all epithelial subsets (**Figure 3E**). Consistently, inferCNV analysis further supported the malignant nature of Cancer-AT2 cells by demonstrating more pronounced copy number variation patterns than those observed in other epithelial populations (**Figure 3F**). We next examined the expression of representative epithelial marker genes across these subpopulations. CEACAM6, HPGD, SFTPA1, SFTPD, AGER, SCGB3A2, CAPS, and KRT17 showed clear subtype-specific expression patterns (**Figure 3G**). CEACAM6 was mainly expressed in Cancer-AT2, AT1, and Club cells, whereas HPGD was largely restricted to Cancer-AT2 cells. SFTPA1 and SFTPD were enriched in Cancer-AT2, Normal-AT2, AT1, and Club cells, while AGER was predominantly confined to AT1 cells. SCGB3A2 was detected mainly in Cancer-AT2, Normal-AT2, and Club cells, CAPS was enriched in Club, Ciliated, and Basal cells, and KRT17 was primarily expressed in Basal cells (**Figure 3H**). Together, these findings define a heterogeneous epithelial landscape in MIA and identify Cancer-AT2 as the predominant malignant epithelial subpopulation.

### 3.5 Heterogeneity of fibroblast-lineage cells and enrichment of POSTN^+^ CAFs in MIA

**Figure 4:**
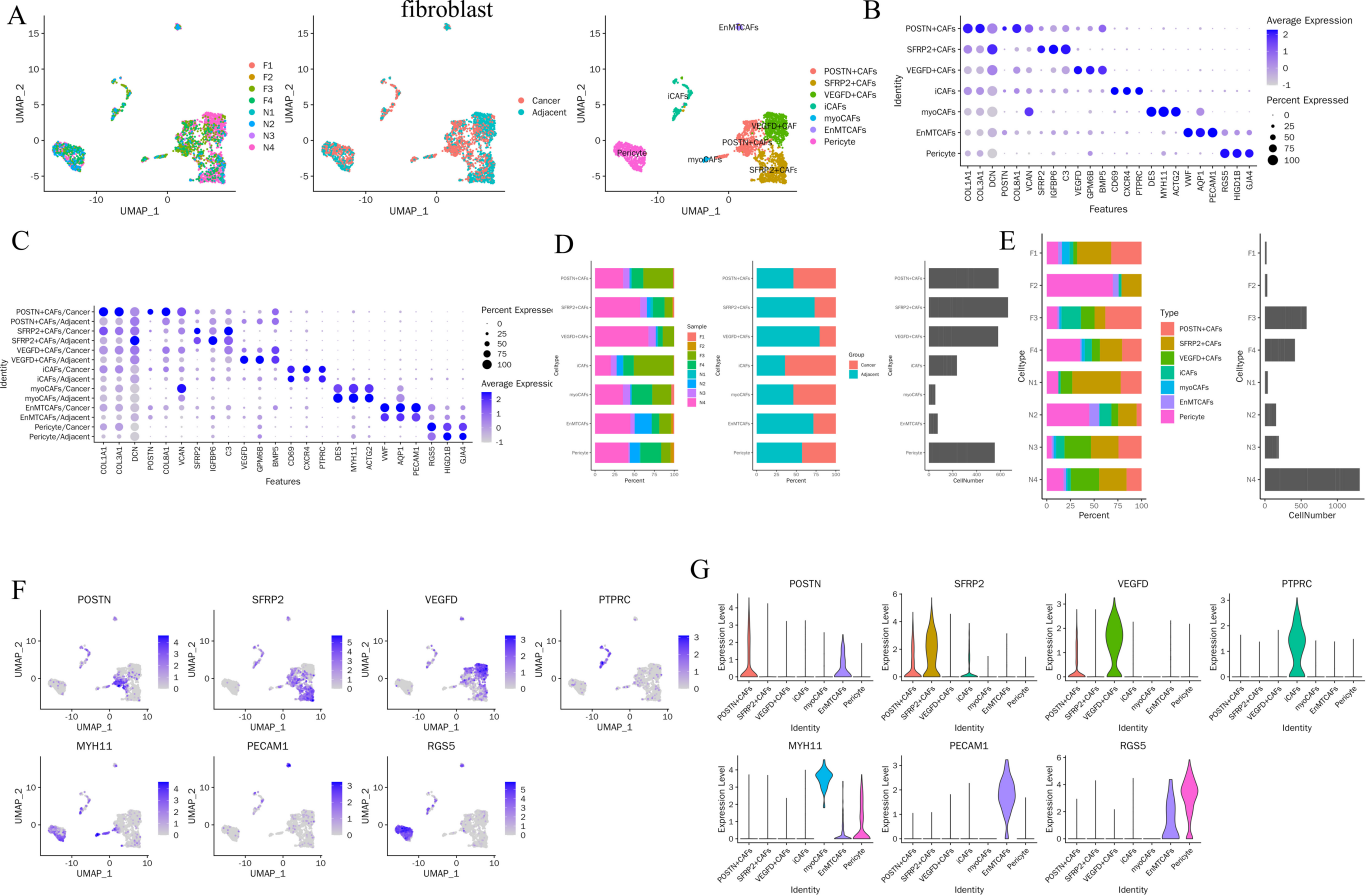
Heterogeneity of fibroblast-lineage cells in MIA and enrichment of POSTN^+^ CAFs in tumor tissues. **(A)** Fibroblasts were divided into 7 subsets: POSTN+CAFs, SFRP2+CAFs, VEGFD+CAFs, iCAFs, myoCAFs, EnMTCAFs, Pericyte. **(B)** Canonical marker genes for fibroblast subtype annotation shown as a dot plot. **(C)** Comparison of subtype-associated marker expression between cancer and adjacent tissues. **(D, E)** Summary of fibroblasts cell subset proportions in carcinoma and adjacent tissues. **(F)** Feature plots of representative marker genes across fibroblast-lineage cells. **(G)** Violin plots showing subtype-specific expression of representative marker genes.

To characterize fibroblast heterogeneity within the MIA microenvironment, fibroblast-lineage cells were further resolved into seven CAF subtypes, including POSTN^+^ CAFs, SFRP2^+^ CAFs, VEGFD^+^ CAFs, inflammatory CAFs (iCAFs), myofibroblastic CAFs (myoCAFs), endothelial–mesenchymal transition CAFs (EnMTCAFs), and pericytes (**Figures 4A–C**). Among these populations, POSTN^+^ CAFs, SFRP2^+^ CAFs, VEGFD^+^ CAFs, and pericytes constituted the predominant fractions (**Figure 4D**). The proportions of iCAFs (69.26% vs. 30.74%), POSTN^+^ CAFs (65.6% vs. 34.4%), and myoCAFs (53.7% vs. 46.3%) were markedly higher in tumor tissues than in adjacent tissues (**Figure 4E**), indicating a preferential expansion of these activated fibroblast subsets in the MIA stroma. Consistent with their annotations, POSTN, SFRP2, VEGFD, PTPRC, MYH11, PECAM1, and RGS5 were among the most prominently upregulated genes in fibroblast-lineage cells (**Figure 4F**). Their expression displayed clear subset specificity: POSTN was mainly confined to POSTN^+^ CAFs and EnMTCAFs; SFRP2 to POSTN^+^ CAFs and SFRP2^+^ CAFs; VEGFD to POSTN^+^ CAFs and VEGFD^+^ CAFs; PTPRC to iCAFs; MYH11 to myoCAFs, EnMTCAFs, and pericytes; PECAM1 to EnMTCAFs; and RGS5 to EnMTCAFs and pericytes (**Figure 4G**). These findings define a highly heterogeneous fibroblast compartment in MIA and identify POSTN^+^ CAFs as a transcriptionally distinct and tumor-enriched stromal population.

### 3.6 MIA tissue-derived exosomes carry a distinct, ECM-related mRNA cargo

Exosomes isolated from MIA lesions and matched adjacent tissues displayed typical discoid morphology under transmission electron microscopy and expressed classical exosomal markers CD63, CD81, and TSG101, confirming the successful isolation of bona fide exosomes (**Supplementary Figures 4A, B**). Comparative transcriptomic profiling revealed that exosomes from MIA tissues contained a distinct mRNA cargo compared with those derived from matched adjacent tissues, comprising 588 differentially expressed mRNAs, of which 212 were upregulated and 376 were downregulated (**Supplementary Figures 5A, B**). Among the upregulated transcripts, CCL19, NHS, URB1, PHLDA2, and POSTN showed the greatest increases in expression. Functional enrichment analysis indicated that differentially expressed exosomal mRNAs were mainly associated with basic cellular and regulatory processes at the Gene Ontology (GO) level (**Supplementary Figure 5C**), whereas KEGG pathway analysis highlighted significant enrichment in the Fanconi anemia pathway, ECM–receptor interaction, and other related signaling pathways (**Supplementary Figure 5D**). Together, these results indicate that tissue-derived exosomes in MIA possess a remodeled mRNA landscape enriched in ECM-associated transcripts, including POSTN, which may contribute to tumor–stroma crosstalk and microenvironmental remodeling.

### 3.7 Fibroblasts show the strongest association with tissue-derived exosomal signatures in MIA

**Figure 5:**
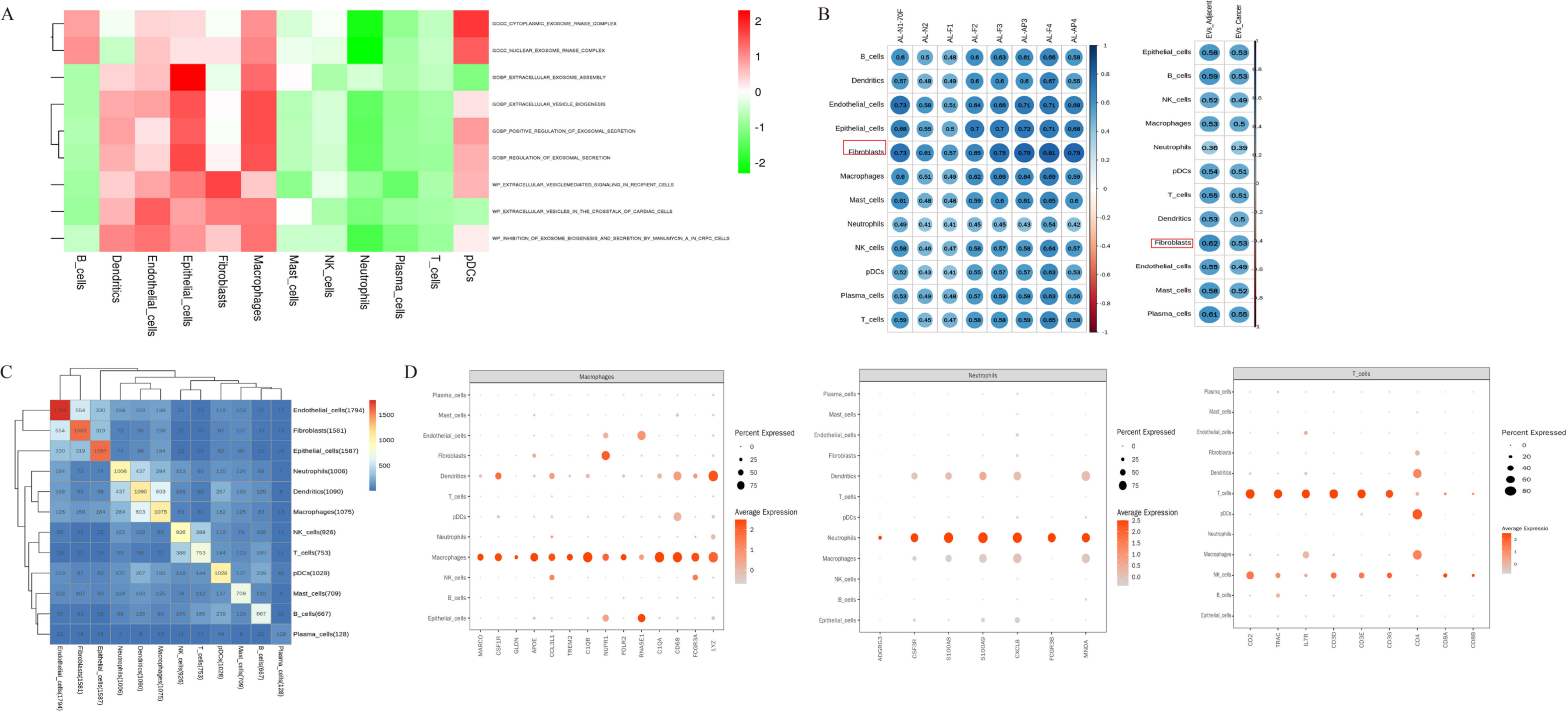
Integrated analysis of tissue-derived exosomes and single-cell transcriptomes identifies fibroblasts as the cell population most closely associated with exosomal signatures in MIA. **(A)** Heatmap showing the enrichment of exosome-related pathways across major cell populations. **(B)** Bubble plot showing the correlations between major cell populations and tissue-derived exosomal transcriptomic profiles. **(C)** Pairwise overlap matrix of highly expressed genes among major cell populations. **(D)** Dot plots showing the expression of representative macrophage-associated genes, neutrophil-associated genes, and T-cell-associated genes across major cell populations. Dot size represents the percentage of cells expressing each gene, and color intensity indicates the average expression level.

To identify the cell population most closely associated with tissue-derived exosomal signatures in MIA, we integrated exosomal and single-cell transcriptomic data. Enrichment analysis of exosome-related pathways showed that fibroblasts, epithelial cells, macrophages, endothelial cells, and dendritic cells had the strongest representation of exosome-associated programs among the major cell populations (**Figure 5A**). Correlation analysis further showed that fibroblasts had the strongest and most consistent association with exosomal transcriptomic profiles across individual exosome samples, and this relationship remained evident at the group level (**Figure 5B**). We next examined the overlap of highly expressed genes across major cell populations. Endothelial cells, fibroblasts, and epithelial cells shared a substantial number of transcripts, while neutrophils, dendritic cells, and macrophages also showed considerable overlap; NK cells and T cells displayed a similarly related expression pattern (**Figure 5C**). In line with these findings, endothelial cells, fibroblasts, and epithelial cells contained the largest numbers of highly expressed genes among the major cell populations. In addition, representative marker-gene analysis further supported the presence of shared transcriptional features across related cell types (**Figure 5D**). Tese results indicate that fibroblasts are most closely linked to tissue-derived exosomal signatures in MIA, while exosome-associated transcriptional features are shared across several stromal and immune cell populations.

### 3.8 Enhanced epithelial–fibroblast crosstalk centered on cancer-AT2 cells and POSTN^+^ CAFs in MIA

**Figure 6:**
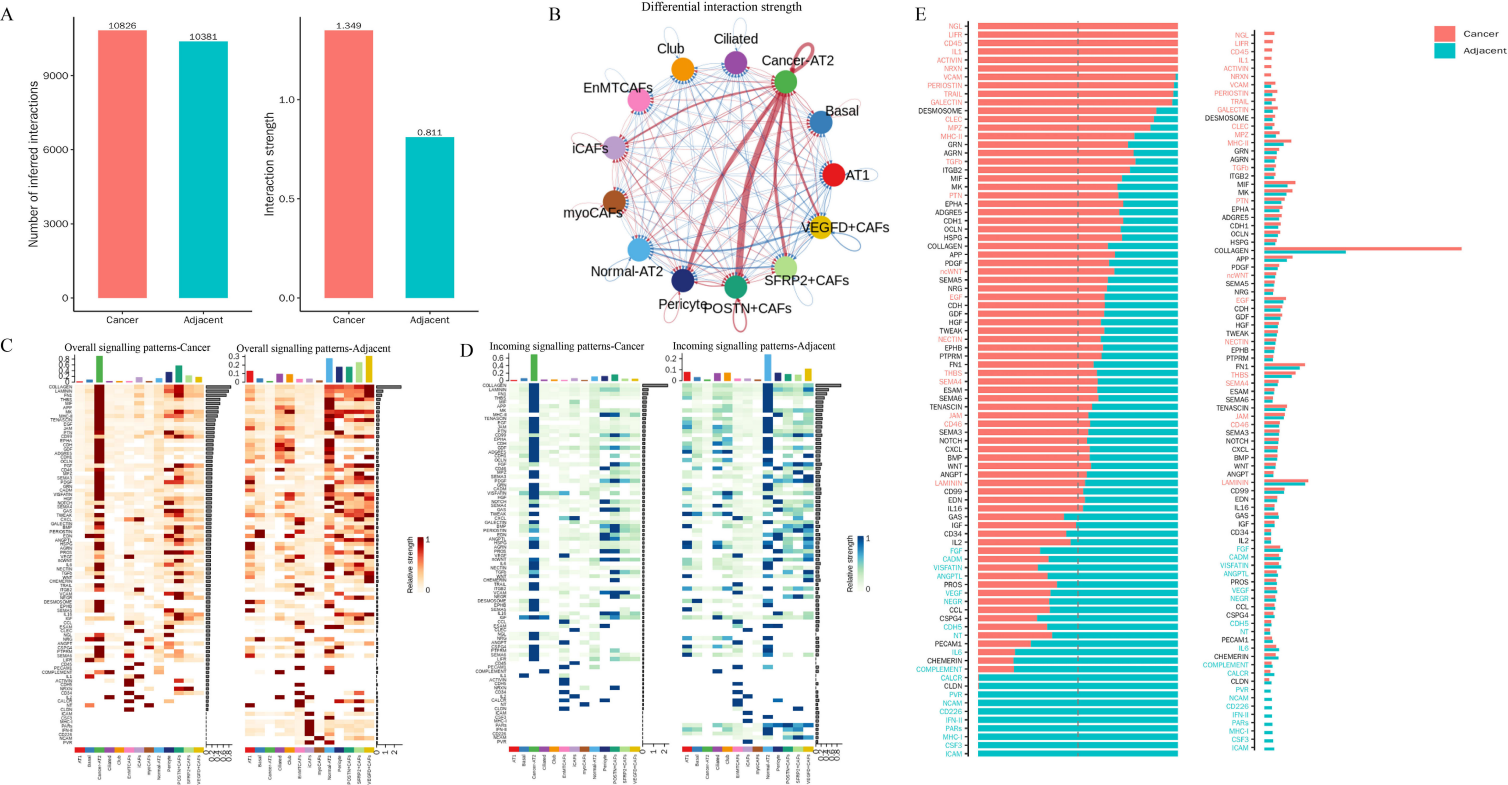
Enhanced epithelial–fibroblast crosstalk centered on cancer-AT2 cells and POSTN^+^ CAFs in MIA. **(A)** Bar plots showing the total number of inferred interactions and the overall interaction strength between epithelial and fibroblast compartments in cancer and adjacent tissues. **(B)** Differential interaction network among epithelial and fibroblast subpopulations. **(C)** Heatmaps showing overall signaling patterns of epithelial and fibroblast subpopulations in cancer and adjacent tissues. **(D)** Heatmaps showing incoming signaling patterns of epithelial and fibroblast subpopulations in cancer and adjacent tissues. **(E)** Molecules unique to tumor or adjacent tissue in epithelial-fibroblast communication.

To characterize epithelial–fibroblast communication in MIA, we compared inferred interactions between epithelial and fibroblast subpopulations in tumor and matched adjacent tissues. Tumor tissues showed a slightly greater number of inferred interactions than adjacent tissues (10, 826 vs. 10, 381), together with a substantially higher overall interaction strength (1, 349 vs. 811; **Figure 6A**), indicating enhanced crosstalk between these two compartments in the tumor microenvironment. Communication network analysis showed that among epithelial subsets, Cancer-AT2 cells engaged most frequently with fibroblast populations, with the strongest interactions observed with POSTN^+^ CAFs, followed by SFRP2^+^ CAFs, VEGFD^+^ CAFs, and pericytes (**Figure 6B**). This hierarchy was consistently observed for both overall signaling and incoming signaling intensity (**Figures 6C, D**). At the pathway level, the communication repertoire differed markedly between the two conditions. Several pathways, including NGL, LIFR, CD45, IL1, ACTIVIN, and NRXN, were more prominent in tumor tissues, whereas PVR, NCAM, CD226, IFN-II, PARs, MHC-I, CSF3, and ICAM were more evident in adjacent tissues (**Figure 6E**). Together, these findings indicate that epithelial–fibroblast communication is selectively strengthened in MIA, with Cancer-AT2 cells and POSTN^+^ CAFs forming a major interaction axis within the tumor microenvironment.

### 3.9 Clinical relevance of exosome-derived candidate genes highlights POSTN as a potential biomarker in LUAD

**Figure 7:**
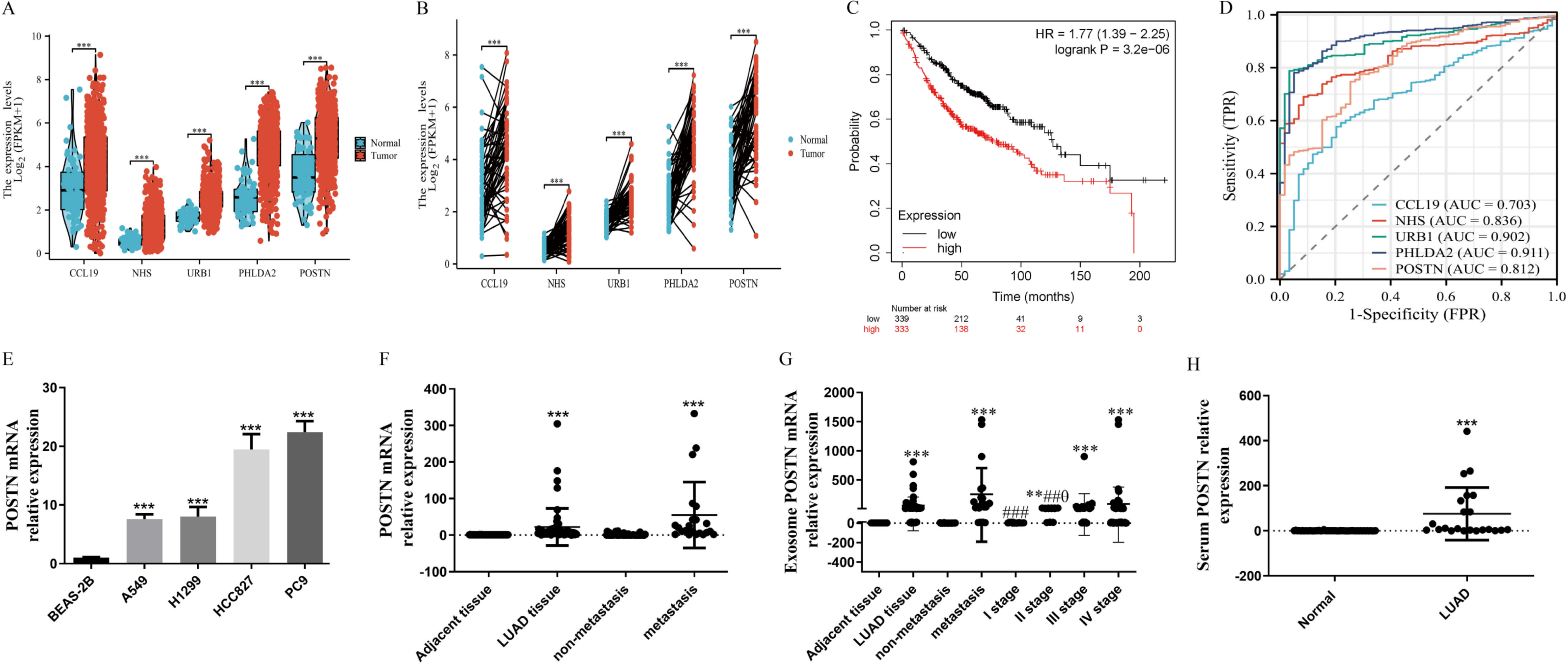
Clinical evaluation of exosome-derived candidate genes and validation of POSTN in LUAD. **(A)** Expression levels of the five most upregulated differentially expressed mRNAs—CCL19, NHS, URB1/KIAA0539, PHLDA2, and POSTN—in tissue-derived exosomes from LUAD tissues and matched adjacent normal tissues. **(B)** Expression levels of CCL19, NHS, URB1, PHLDA2, and POSTN in tumor and normal lung tissues from the TCGA-LUAD cohort. **(C)** Kaplan–Meier overall survival analysis according to POSTN expression in LUAD. **(D)** Receiver operating characteristic **(ROC)** curves showing the diagnostic performance of the five candidate genes in LUAD. **(E)** Relative POSTN mRNA expression in BEAS-2B bronchial epithelial cells and LUAD cell lines (A549, H1299, HCC827, and PC9). **(F)** Relative POSTN mRNA expression in adjacent and LUAD tissues, and in non-metastatic and metastatic LUAD samples. **(G)** Tissue-derived exosomal POSTN mRNA levels in LUAD, stratified by lymph node metastasis status and pathological stage. **(H)** Relative serum exosomal POSTN mRNA expression in healthy controls and patients with LUAD. *Asterisks indicate statistical significance versus adjacent tissue or the non-metastatic group (**P < 0.01; ***P < 0.001), and hash symbols indicate significance between tumor subgroups (^##^*P* < 0.01; ^###^*P* < 0.001).

To assess the clinical relevance of exosome-derived candidate genes, we examined their expression, prognostic value, and diagnostic performance in LUAD, with a particular focus on POSTN. Compared with adjacent normal tissues, LUAD tissue–derived exosomes showed marked upregulation of the five most differentially expressed mRNAs: CCL19 (fold change = 12.7), NHS (fold change = 8.9), URB1/KIAA0539 (fold change = 6.9), PHLDA2 (fold change = 9.5), and POSTN (fold change = 10.2) (**Figure 7A**). In the TCGA-LUAD cohort (n = 598), all five genes were significantly overexpressed in tumor tissues relative to normal lung (**Figure 7B**; P < 0.001). Survival analysis showed that CCL19 was not associated with overall survival, whereas higher NHS expression predicted better prognosis; in contrast, higher URB1, PHLDA2, and POSTN expression was associated with worse overall survival (**Figure 7C**; **Supplementary Figures 6A–D**). ROC analysis indicated that all five genes possessed diagnostic value for LUAD, with AUCs of 0.703 (CCL19), 0.836 (NHS), 0.902 (URB1), 0.911 (PHLDA2), and 0.812 (POSTN) (**Figure 7D**).

Because POSTN was associated with poor prognosis, it was selected for further validation. POSTN mRNA expression was significantly higher in LUAD cell lines than in BEAS-2B cells (**Figure 7E**). In clinical samples, POSTN expression was increased in LUAD tissues relative to adjacent tissues and was higher in metastatic than in non-metastatic cases (**Figure 7F**). A similar pattern was observed for tissue-derived exosomal POSTN, which was elevated in LUAD, associated with metastasis, and varied across clinical stages (**Figure 7G**). In addition, serum exosomal POSTN was significantly increased in patients with LUAD compared with healthy controls (**Figure 7H**). Together, these findings support the clinical relevance of POSTN in LUAD.

### 3.10 POSTN^+^ CAF-derived exosomes promote LUAD cell malignancy and tumor growth

To clarify the source and functional significance of exosomal POSTN in the MIA/LUAD microenvironment, we combined transcriptomic analyses with *in vitro* and *in vivo* experiments. Immune infiltration analysis showed that POSTN expression was negatively correlated with tumor purity and B-cell infiltration, but positively correlated with CD8^+^ T cells, macrophages, neutrophils, and dendritic cells (**Supplementary Figure 7A**), indicating that elevated POSTN expression is associated with a stromal- and immune-enriched tumor microenvironment. Consistent with this observation, analysis of the NSCLC_GSE127465 and NSCLC_GSE148071 datasets showed that POSTN expression was highest in fibroblasts among tumor microenvironment–associated cell populations (**Supplementary Figure 7B**). Together, these findings support fibroblasts, particularly POSTN^+^ CAFs, as a major source of POSTN in the tumor microenvironment.

**Figure 8:**
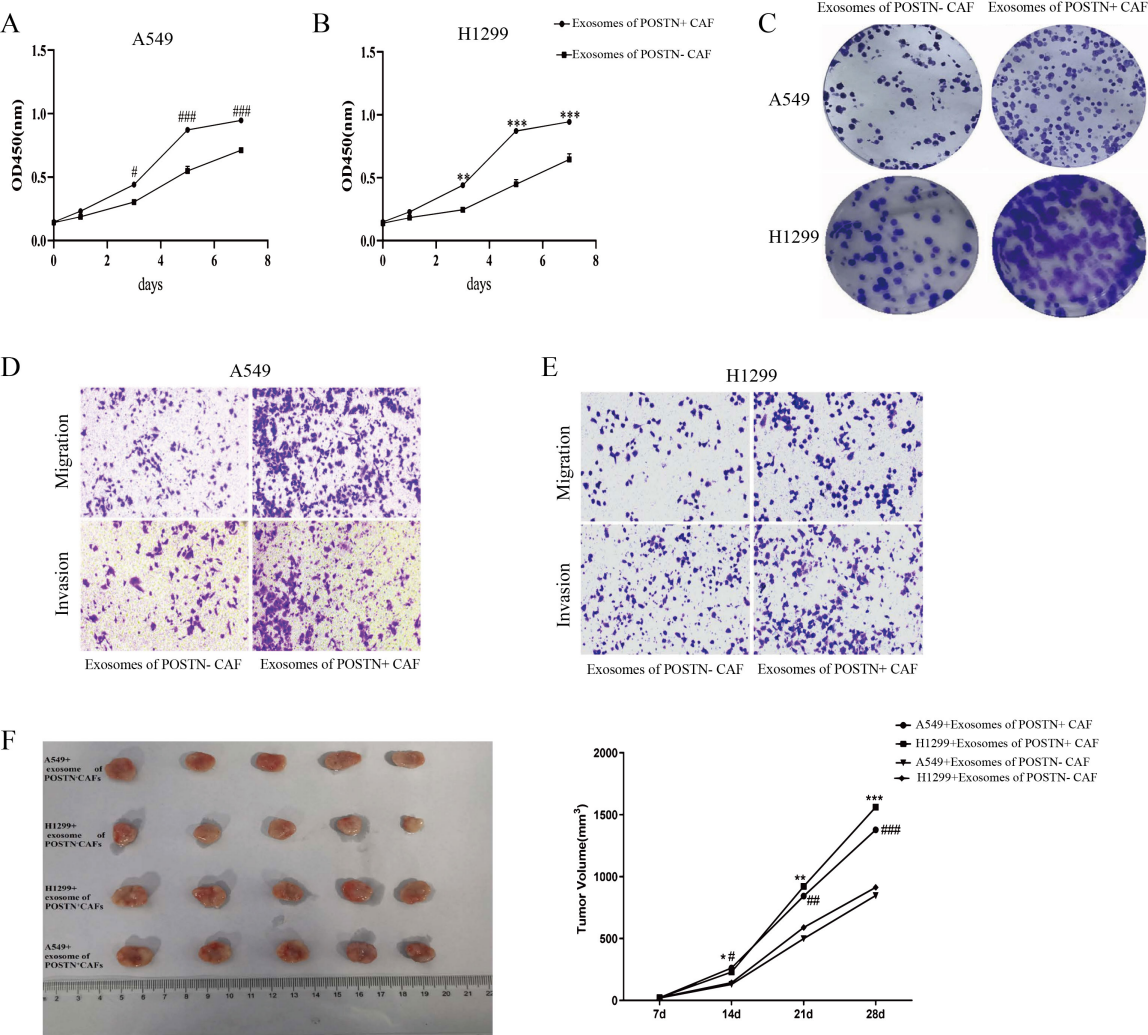
*In vitro* and *in vivo* effects of POSTN^+^ CAF–derived exosomes on LUAD cells. **(A, B)** CCK-8 assays showing the proliferation of A549 **(A)** and H1299 **(B)** cells after treatment with exosomes derived from POSTN^-^ CAFs or POSTN^+^ CAFs. **(C)** Representative images of colony formation in A549 and H1299 cells treated with exosomes from POSTN^-^ CAFs or POSTN^+^ CAFs. **(D, E)** Representative images of Transwell migration and invasion assays in A549 **(D)** and H1299 **(E)** cells treated with exosomes from POSTN^-^ CAFs or POSTN^+^ CAFs. **(F)** Tumor growth in nude mice bearing A549 or H1299 xenografts treated with PBS, POSTN^-^ CAF–derived exosomes, or POSTN^+^ CAF–derived exosomes. *P < 0.05, **P < 0.01, ***P < 0.001 versus the control group; ^#^*P* < 0.05, ^##^*P* < 0.01, ^###^*P* < 0.001 versus the POSTN^-^ CAF–derived exosome group.

We next assessed the functional effects of POSTN^+^ CAF-derived exosomes on LUAD cells. Compared with exosomes from POSTN^-^ CAFs, exosomes derived from POSTN^+^ CAFs significantly promoted the proliferation of both A549 and H1299 cells (**Figures 8A, B**). Colony formation assays showed a similar increase in clonogenic growth after treatment with POSTN^+^ CAF-derived exosomes (**Figure 8C**). In addition, Transwell assays demonstrated that POSTN^+^ CAF-derived exosomes enhanced the migratory and invasive capacities of both LUAD cell lines (**Figures 8D, E**). The tumor-promoting effect of POSTN^+^ CAF-derived exosomes was further confirmed *in vivo*. In xenograft models established with A549 and H1299 cells, mice receiving POSTN^+^ CAF-derived exosomes developed larger tumors than those receiving POSTN^-^ CAF-derived exosomes, and tumor growth was accelerated over time (**Figure 8F**). Collectively, these results indicate that POSTN^+^ CAF-derived exosomes enhance the malignant behavior of LUAD cells and promote tumor growth.

## 4 Discussion

Despite the recognized importance of CAFs and EVs in LUAD (21), how fibroblast-derived exosomal signals contribute to microenvironmental remodeling and early progression in MIA remains insufficiently understood. In the present study, we integrated single-cell transcriptomics, tissue-derived exosomal RNA sequencing, clinical validation, and functional assays to investigate stromal–epithelial communication in microinvasive lung adenocarcinoma. Our data indicate that MIA already exhibits substantial microenvironmental remodeling, characterized by expansion of a malignant Cancer-AT2 epithelial population, enrichment of POSTN^+^ CAFs, and strengthened communication between fibroblasts and epithelial cells. We further found that POSTN was elevated in tissue-derived exosomes and remained clinically relevant across tissue, exosomal, and serum-based analyses. Functionally, exosomes derived from POSTN^+^ CAFs promoted LUAD cell proliferation, migration, invasion, clonogenic growth, and xenograft progression. Taken together, these findings support a model in which POSTN^+^ CAFs represent an active stromal component in early LUAD evolution and suggest that exosomal POSTN may involved in tumour-promoting stromal–epithelial crosstalk.

It is worth noting that POSTN is not a newly identified CAF marker; rather, it has already been recognized as a prominent stromal and CAF-associated molecule in NSCLC. For example, Chen et al. identified a myofibroblastic CAF subpopulation, POSTN^+^ CAFs, which might associate with SPP1^+^ macrophages to promote the formation of desmoplastic architecture and participate in immune suppression (22). In addition, Wasik et al. confirmed that high POSTN expression may be critical for angiogenesis and the progression of NSCLC and serves as an independent prognostic factor for NSCLC (23). Thus, the principal contribution of the present work is not to redefine POSTN as a CAF biomarker, but to extend this framework by showing that POSTN in CAF-derived exosomes is associated with aggressive tumour phenotypes and may participate in early-stage LUAD progression. Our study also provide a plausible biological context for the tumour-promoting effects of POSTN-positive CAF-derived exosomes. Our cell–cell communication analysis highlighted strong epithelial–fibroblast interactions centered on Cancer-AT2 cells and POSTN^+^ CAFs, with enrichment of ECM-related signaling. This observation is consistent with the known biology of POSTN as a matricellular protein involved in matrix organization, cell adhesion, and tumour-supportive stromal activation. Previous studies have shown that CAF-derived POSTN can enhance NSCLC cell proliferation, migration, epithelial–mesenchymal transition, and treatment resistance, and that these effects may involve ERK activation or integrin-associated signaling pathways (24–26). Although our study did not directly dissect the downstream pathway activated by exosomal POSTN in recipient cells, the combined transcriptomic and functional evidence supports the interpretation that POSTN^+^ CAFs contribute to a matrix-remodeling stromal program that favors malignant progression.

At the same time, our findings should not be interpreted as evidence that POSTN is transferred exclusively through exosomes. Intercellular communication within the tumour microenvironment is inherently multifaceted and is mediated through several parallel mechanisms, including soluble cytokines and growth factors, extracellular vesicles of different subclasses, matrix-bound signaling, and direct cell–cell contact (27, 28). This issue is particularly relevant for POSTN, which is a secreted matricellular protein and may exert its biological effects in more than one form (29). In addition to being packaged into extracellular vesicles, POSTN may be released into the extracellular space, incorporated into the surrounding matrix, or act in concert with broader stromal remodeling processes driven by activated fibroblasts (30). As a result, the tumour-promoting effects associated with POSTN^+^ CAFs *in vivo* are likely to reflect a combination of vesicle-mediated transfer and non-vesicular stromal mechanisms rather than a single mode of delivery. Our functional data clearly show that exosomes derived from POSTN^+^ CAFs are sufficient to enhance malignant phenotypes in LUAD cells, supporting exosomes as a biologically relevant route of stromal signaling. However, these experiments do not establish that exosomal transfer is the only mechanism responsible for the observed effects, nor do they exclude the contribution of soluble POSTN or other fibroblast-derived factors carried by the same microenvironment. Thus, exosomes represent an important and experimentally supported route through which POSTN-associated stromal signals can be conveyed in MIA, while additional complementary mechanisms are also likely to participate in shaping tumour behavior *in vivo*.

Compared with circulating vesicles, tissue-derived extracellular vesicle preparations are more likely to reflect local tumour–stroma communication because they are obtained directly from the lesion and its surrounding microenvironment and therefore retain more tissue-specific biological information (19, 31). However, extracellular vesicle isolation from solid tissues remains technically challenging. Previous methodological studies have shown that tissue dissociation and ultracentrifugation can effectively enrich vesicle populations, but recovery efficiency, yield, and subtype purity are all influenced by the nature of the starting material and the details of the isolation workflow (19, 31). In addition, growing evidence suggests that tissue-derived preparations may contain heterogeneous extracellular vesicle subpopulations, which should be taken into account when interpreting downstream molecular profiles (32). Consistent with this, the MISEV2018 guidelines recommend that, unless rigorous evidence for vesicle subtype assignment is available, such preparations should be described as enriched extracellular vesicle fractions rather than definitive exosome isolates (33). In our study, vesicle morphology was confirmed by transmission electron microscopy, and the preparations expressed canonical extracellular vesicle markers, including TSG101, CD63, and CD81, supporting the overall quality of the isolated material. Nevertheless, the present data are most appropriately interpreted as arising from enriched tissue-derived extracellular vesicle preparations rather than absolutely pure exosomes.

The clinical observations in this study further support the relevance of POSTN in LUAD. In the TCGA-LUAD cohort, all five exosome-derived candidates were overexpressed in tumors, but only URB1, PHLDA2, and POSTN were clearly associated with poor survival, and POSTN showed strong diagnostic performance. Our own cohort confirmed that POSTN mRNA and exosomal POSTN levels were elevated in LUAD cell lines, tumor tissues, and tissue-derived exosomes, with higher levels in patients with lymph node metastasis and a stepwise increase across pathological stages. Serum exosomal POSTN was also significantly higher in LUAD patients than in healthy controls, echoing previous reports that circulating exosomal POSTN is linked to tumor burden and metastatic behavior in other cancers (34, 35). Importantly, our study extends these observations by tracing POSTN to a specific stromal source and linking it to a biologically meaningful route of communication in an early-stage disease context. In this sense, exosomal POSTN may be considered not only a candidate biomarker, but also a readout of stromal activation during the transition from minimally invasive to more aggressive disease.

This study has several limitations that should be acknowledged. First, the discovery cohort used for single-cell and tissue-derived exosomal profiling was relatively small and derived from a single center, which may limit generalizability; future studies should therefore include larger, multicenter cohorts to confirm the robustness of the observed stromal–exosomal patterns. Second, although our functional assays showed that POSTN^+^ CAF-derived exosomes promote malignant phenotypes, the study did not directly establish that POSTN itself is the sole or indispensable effector; this question should be addressed in future work through targeted POSTN knockdown or overexpression in CAFs, together with rescue experiments in recipient cells. Third, the downstream signaling events triggered by POSTN-associated exosomal transfer were not mechanistically dissected; more detailed pathway analyses, including transcriptomic, proteomic, and inhibition-based validation, will be needed to define the molecular programs activated in recipient tumour cells. Fourth, although our single-cell data strongly suggested enrichment of POSTN^+^ CAFs in the stromal compartment, we were unable to validate their spatial relationship to epithelial regions, including the Cancer-AT2 compartment, by immunofluorescence, immunohistochemistry, or spatial transcriptomic approaches; future studies incorporating these methods would help define the anatomical distribution of POSTN^+^ CAFs and clarify whether their localisation supports the proposed stromal–epithelial communication axis. Fifth, because intercellular transfer within the tumour microenvironment is not limited to exosomes, the current study cannot exclude contributions from soluble factors, other extracellular vesicle subtypes, or contact-dependent mechanisms; future experiments using more selective vesicle isolation strategies, secretion-blocking approaches, and conditioned-medium controls would help distinguish these possibilities. Finally, although the extracellular vesicle preparations were validated morphologically and by canonical markers, tissue-derived vesicle isolation remains technically challenging and may yield heterogeneous vesicle populations; accordingly, future studies should incorporate more rigorous extracellular vesicle characterization and standardized isolation workflows to improve purity, reproducibility, and cell-of-origin resolution.

## 5 Conclusion

In summary, our work reveals that even at the microinvasive stage, LUAD is characterized by a profoundly remodeled microenvironment in which POSTN^+^ CAFs emerge as central stromal players. By integrating single-cell and tissue-derived exosomal transcriptomes with functional experiments, we identify a POSTN^+^ CAF–exosomal POSTN–Cancer-AT2 axis that enhances malignant epithelial behavior and is closely linked to disease progression and poor prognosis. These findings not only provide mechanistic insight into how stromal fibroblasts and exosomes drive MIA evolution but also highlight exosomal POSTN as a promising biomarker and potential therapeutic target in early-stage LUAD. Future studies targeting POSTN^+^ CAFs or disrupting exosomal POSTN signaling, alone or in combination with existing targeted and immunotherapies, may offer new avenues to prevent or delay the progression of MIA to invasive lung adenocarcinoma.

## Data Availability

The publicly available datasets analyzed in this study were obtained from The Cancer Genome Atlas Lung Adenocarcinoma cohort (TCGA-LUAD; https://portal.gdc.cancer.gov/projects/TCGA-LUAD) and the Gene Expression Omnibus database under accession numbers GSE127465 (https://www.ncbi.nlm.nih.gov/geo/query/acc.cgi?acc=GSE127465) and GSE148071 (https://www.ncbi.nlm.nih.gov/geo/query/acc.cgi?acc=GSE148071). The single-cell RNA sequencing and tissue-derived exosomal RNA sequencing data generated from patient-derived clinical specimens in the present study are not publicly deposited because they contain potentially sensitive human transcriptomic information, and public release of the raw sequencing data was not covered by the scope of the informed consent and institutional ethical approval. De-identified data supporting the findings of this study are available from the corresponding author upon reasonable request, subject to institutional approval and appropriate data-use agreements.

## Glossary

NSCLC: Non-small cell lung cancer
MIA: Microinvasive adenocarcinoma
LUAD: Lung adenocarcinoma
CAFs: Cancer-associated fibroblasts
POSTN: Periostin
TME: Tumor microenvironment
scRNA-seq: Single-cell RNA sequencing
TCGA: The Cancer Genome Atlas
GEO: Gene Expression Omnibus
GO: Gene Ontology
KEGG: Kyoto Encyclopedia of Genes and Genomes
ROC: Receiver Operating Characteristic
ECM: Extracellular matrix
EMT: Epithelial-mesenchymal transition
EVs: Extracellular vehicles
BCA: Bicinchoninic acid
CCK-8: Cell Counting Kit-8
PBS: Phosphate-buffered saline
RT-qPCR: Reverse transcription quantitative polymerase chain reaction
SPF: Specific pathogen-free
NK cells: Natural killer cells
pDCs: Plasmacytoid dendritic cells
AT2: Alveolar type II
iCAFs: Inflammatory cancer-associated fibroblasts
myoCAFs: Myofibroblastic cancer-associated fibroblasts
EnMTCAFs: Endothelial–mesenchymal transition cancer-associated fibroblasts
AUC: Area under the curve
CNV: Copy number variation
GSVA: Gene set variation analysis
LR-pairs: Ligand–receptor pairs
FDR: False Discovery Rate
OS: Overall survival

## References

[1] Sung H Ferlay J Siegel RL Laversanne M Soerjomataram I Jemal A . Global cancer statistics 2020: GLOBOCAN estimates of incidence and mortality worldwide for 36 cancers in 185 countries. CA Cancer J Clin. (2021) 71:209–49. doi: 10.3322/caac.21660. PMID: 33538338

[2] Shi J Shiraishi K Choi J Matsuo K Chen TY Dai J . Genome-wide association study of lung adenocarcinoma in East Asia and comparison with a European population. Nat Commun. (2023) 14:3043. doi: 10.1038/s41467-023-38196-z. PMID: 37236969 PMC10220065

[3] Kim DH Park S Kim H Choi YJ Kim SY Sung KJ . Tumor-derived exosomal miR-619-5p promotes tumor angiogenesis and metastasis through the inhibition of RCAN1.4. Cancer Lett. (2020) 475:2–13. doi: 10.1016/j.canlet.2020.01.023. PMID: 32004570

[4] Yotsukura M Asamura H Motoi N Kashima J Yoshida Y Nakagawa K . Long-term prognosis of patients with resected adenocarcinoma in situ and minimally invasive adenocarcinoma of the lung. J Thorac Oncol. (2021) 16:1312–20. doi: 10.1016/j.jtho.2021.04.007. PMID: 33915249

[5] Inamura K . Clinicopathological characteristics and mutations driving development of early lung adenocarcinoma: Tumor initiation and progression. Int J Mol Sci. (2018) 19:1259. doi: 10.3390/ijms19041259. PMID: 29690599 PMC5979290

[6] Zhang W Xu H Tang N Han S Shu H . Genomic landscape features of minimally invasive adenocarcinoma and invasive lung adenocarcinoma. Glob Med Genet. (2024) 11:312–8. doi: 10.1055/s-0044-1791198. PMID: 39583122 PMC11412754

[7] Valadi H Ekström K Bossios A Sjöstrand M Lee JJ Lötvall JO . Exosome-mediated transfer of mRNAs and microRNAs is a novel mechanism of genetic exchange between cells. Nat Cell Biol. (2007) 9:654–9. doi: 10.1038/ncb1596. PMID: 17486113

[8] Nie H Xie X Zhang D Zhou Y Li B Li F . Use of lung-specific exosomes for miRNA-126 delivery in non-small cell lung cancer. Nanoscale. (2020) 12:877–87. doi: 10.1039/c9nr09011h. PMID: 31833519

[9] Li Q . Role of exosomes in cellular communication between tumor cells and the tumor microenvironment. Oncol Lett. (2022) 24:240. doi: 10.3892/ol.2022.13360. PMID: 35720493 PMC9185148

[10] Jeurissen S Vergauwen G Van Deun J Lapeire L Depoorter V Miinalainen I . The isolation of morphologically intact and biologically active extracellular vesicles from the secretome of cancer-associated adipose tissue. Cell Adh Migr. (2017) 11:196–204. doi: 10.1080/19336918.2017.1279784. PMID: 28146372 PMC5351718

[11] Hoshino A Kim HS Bojmar L Gyan KE Cioffi M Hernandez J . Extracellular vesicle and particle biomarkers define multiple human cancers. Cell. (2020) 182:1044–1061.e1018. doi: 10.1016/j.cell.2020.07.009. PMID: 32795414 PMC7522766

[12] Domcke S Sinha R Levine DA Sander C Schultz N . Evaluating cell lines as tumour models by comparison of genomic profiles. Nat Commun. (2013) 4:2126. doi: 10.1038/ncomms3126. PMID: 23839242 PMC3715866

[13] Chen B Sirota M Fan-Minogue H Hadley D Butte AJ . Relating hepatocellular carcinoma tumor samples and cell lines using gene expression data in translational research. BMC Med Genomics. (2015) 8:S5. doi: 10.1186/1755-8794-8-s2-s5. PMID: 26043652 PMC4460709

[14] Sun B Xun Z Zhang N Liu K Chen X Zhao H . Single-cell RNA sequencing in cancer research: discovering novel biomarkers and therapeutic targets for immune checkpoint blockade. Cancer Cell Int. (2023) 23:313. doi: 10.1186/s12935-023-03158-4. PMID: 38066642 PMC10704754

[15] Qian J Olbrecht S Boeckx B Vos H Laoui D Etlioglu E . A pan-cancer blueprint of the heterogeneous tumor microenvironment revealed by single-cell profiling. Cell Res. (2020) 30:745–62. doi: 10.1038/s41422-020-0355-0. PMID: 32561858 PMC7608385

[16] Bischoff P Trinks A Obermayer B Pett JP Wiederspahn J Uhlitz F . Single-cell RNA sequencing reveals distinct tumor microenvironmental patterns in lung adenocarcinoma. Oncogene. (2021) 40:6748–58. doi: 10.1038/s41388-021-02054-3. PMID: 34663877 PMC8677623

[17] Wang R Xu Y Tong L Zhang X Zhang S . Recent progress of exosomal lncRNA/circRNA-miRNA-mRNA axis in lung cancer: implication for clinical application. Front Mol Biosci. (2024) 11:1417306. doi: 10.3389/fmolb.2024.1417306. PMID: 39021878 PMC11251945

[18] Lin S Zhou S Han X Yang Y Zhou H Chang X . Single-cell analysis reveals exosome-associated biomarkers for prognostic prediction and immunotherapy in lung adenocarcinoma. Aging (Albany Ny). (2023) 15:11508–31. doi: 10.18632/aging.205140. PMID: 37878007 PMC10637798

[19] Vella LJ Scicluna BJ Cheng L Bawden EG Masters CL Ang CS . A rigorous method to enrich for exosomes from brain tissue. J Extracell Vesicles. (2017) 6:1348885. doi: 10.1080/20013078.2017.1348885. PMID: 28804598 PMC5533148

[20] Théry C Amigorena S Raposo G Clayton A . Isolation and characterization of exosomes from cell culture supernatants and biological fluids. Curr Protoc Cell Biol. (2006) Chapter 3:Unit 3.22. doi: 10.1002/0471143030.cb0322s30. PMID: 18228490

[21] LeBleu VS Kalluri R . A peek into cancer-associated fibroblasts: origins, functions and translational impact. Dis Models Mech. (2018) 11:355–68. doi: 10.1242/dmm.029447. PMID: 29686035 PMC5963854

[22] Chen C Guo Q Liu Y Hou Q Liao M Guo Y . Single-cell and spatial transcriptomics reveal POSTN(+) cancer-associated fibroblasts correlated with immune suppression and tumour progression in non-small cell lung cancer. Clin Transl Med. (2023) 13:e1515. doi: 10.1002/ctm2.1515. PMID: 38115703 PMC10731139

[23] Wasik A Podhorska-Okolow M Dziegiel P Piotrowska A Kulus MJ Kmiecik A . Correlation between periostin expression and pro-angiogenic factors in non-small-cell lung carcinoma. Cells. (2024) 13:793–807. doi: 10.3390/cells13171406. PMID: 39272978 PMC11394527

[24] Takatsu F Suzawa K Tomida S Thu YM Sakaguchi M Toji T . Periostin secreted by cancer-associated fibroblasts promotes cancer progression and drug resistance in non-small cell lung cancer. J Mol Med (Berl). (2023) 101:1603–14. doi: 10.1007/s00109-023-02384-7. PMID: 37831111

[25] Yamato H Kimura K Fukui E Kanou T Ose N Funaki S . Periostin secreted by activated fibroblasts in idiopathic pulmonary fibrosis promotes tumorigenesis of non-small cell lung cancer. Sci Rep. (2021) 11:21114. doi: 10.1038/s41598-021-00717-5. PMID: 34702952 PMC8548404

[26] Jin X Deng Q Ye S Liu S Fu Y Liu Y . Cancer-associated fibroblast-derived periostin promotes papillary thyroid tumor growth through integrin-FAK-STAT3 signaling. Theranostics. (2024) 14:3014–28. doi: 10.7150/thno.94207. PMID: 38773979 PMC11103496

[27] Maacha S Bhat AA Jimenez L Raza A Haris M Uddin S . Extracellular vesicles-mediated intercellular communication: roles in the tumor microenvironment and anti-cancer drug resistance. Mol Cancer. (2019) 18:55. doi: 10.1186/s12943-019-0965-7. PMID: 30925923 PMC6441157

[28] Maia J Caja S Strano Moraes MC Couto N Costa-Silva B . Exosome-based cell-cell communication in the tumor microenvironment. Front Cell Dev Biol. (2018) 6:18. doi: 10.3389/fcell.2018.00018. PMID: 29515996 PMC5826063

[29] Conway SJ Izuhara K Kudo Y Litvin J Markwald R Ouyang G . The role of periostin in tissue remodeling across health and disease. Cell Mol Life Sciences: CMLS. (2014) 71:1279–88. doi: 10.1007/s00018-013-1494-y. PMID: 24146092 PMC3949008

[30] Kudo A . Periostin in fibrillogenesis for tissue regeneration: periostin actions inside and outside the cell. Cell Mol Life Sciences: CMLS. (2011) 68:3201–7. doi: 10.1007/s00018-011-0784-5. PMID: 21833583 PMC3173633

[31] Hurwitz SN Olcese JM Meckes DG . Extraction of extracellular vesicles from whole tissue. J Vis Exp. (2019) (144):10.3791/59143. doi: 10.3791/59143. PMID: 30799860 PMC7098067

[32] Lin J Lu W Huang B Yang W Wang X . The role of tissue-derived extracellular vesicles in tumor microenvironment. Tissue Cell. (2024) 89:102470. doi: 10.1016/j.tice.2024.102470. PMID: 39002287

[33] Ten A Kumeiko V Farniev V Gao H Shevtsov M . Tumor microenvironment modulation by cancer-derived extracellular vesicles. Cells. (2024) 13:23335–45. doi: 10.3390/cells13080682. PMID: 38667297 PMC11049026

[34] Chen Y Zhang F Zhang B Trojanowicz B Hämmerle M Kleeff J . Periostin is associated with prognosis and immune cell infiltration in pancreatic adenocarcinoma based on integrated bioinformatics analysis. Cancer Rep (Hoboken). (2024) 7:e1990. doi: 10.1002/cnr2.1990. PMID: 38389400 PMC10884618

[35] Silvers CR Liu YR Wu CH Miyamoto H Messing EM Lee YF . Identification of extracellular vesicle-borne periostin as a feature of muscle-invasive bladder cancer. Oncotarget. (2016) 7:23335–45. doi: 10.18632/oncotarget.8024. PMID: 26981774 PMC5029630

