# Supplementary material for: Exosomal POSTN from cancer-associated fibroblasts drives progression of microinvasive lung adenocarcinoma: insights from single-cell and tissue exosome sequencing analysis
Source: Front Immunol. 2026 May 19;17:1767771. doi: 10.3389/fimmu.2026.1767771 (PMC13226168; doi:10.3389/fimmu.2026.1767771)
Supplement: Supplementary file 1 [file Table1.docx]

Supplementary Material

## Supplementary Figure


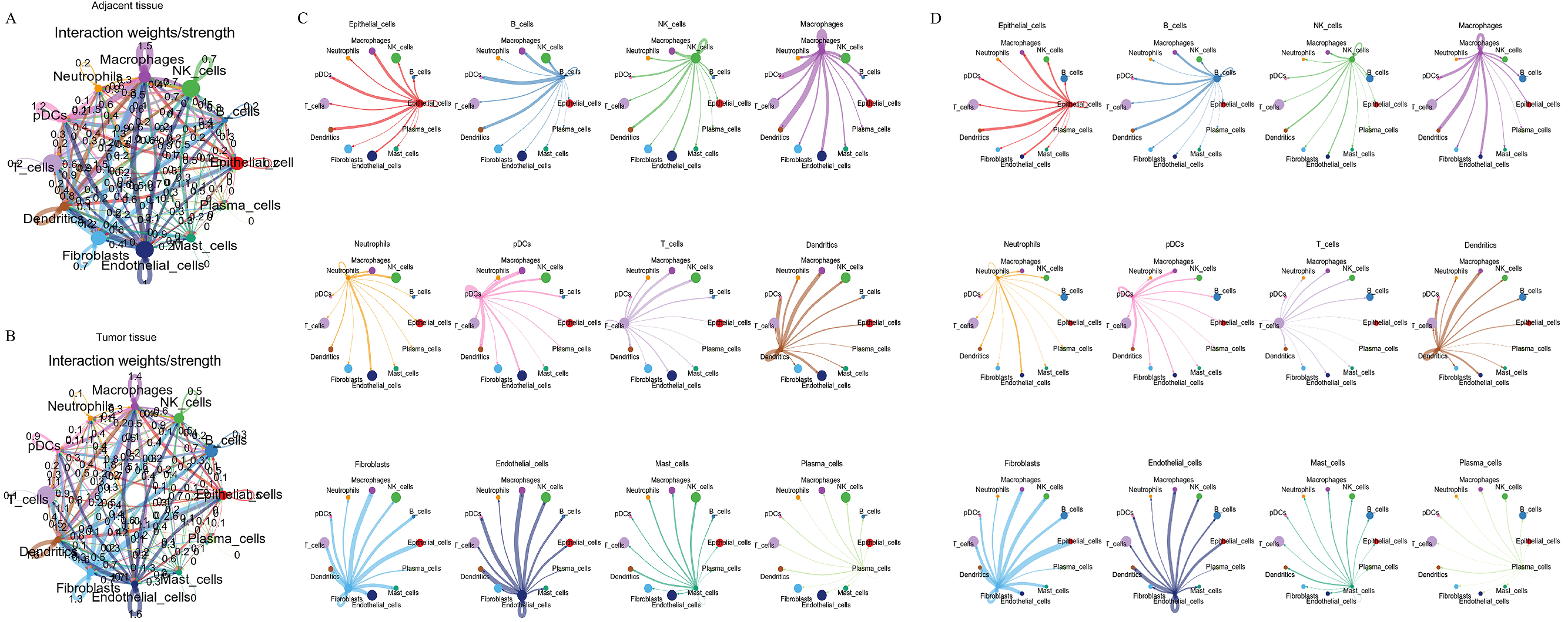


**Supplementary Figure 1.** Global cell–cell communication networks in MIA and matched adjacent tissues. (A) The overall interaction weights/strength among major cell populations in adjacent tissues. (B) The overall interaction weights/strength among major cell populations in MIA tissues. (C) Sender-centered communication networks for individual cell populations in adjacent tissues. (D) Sender-centered communication networks for individual cell populations in MIA tissues. Node size reflects the total interaction strength of each cell population, and edge width indicates the aggregated communication strength inferred by CellChat.


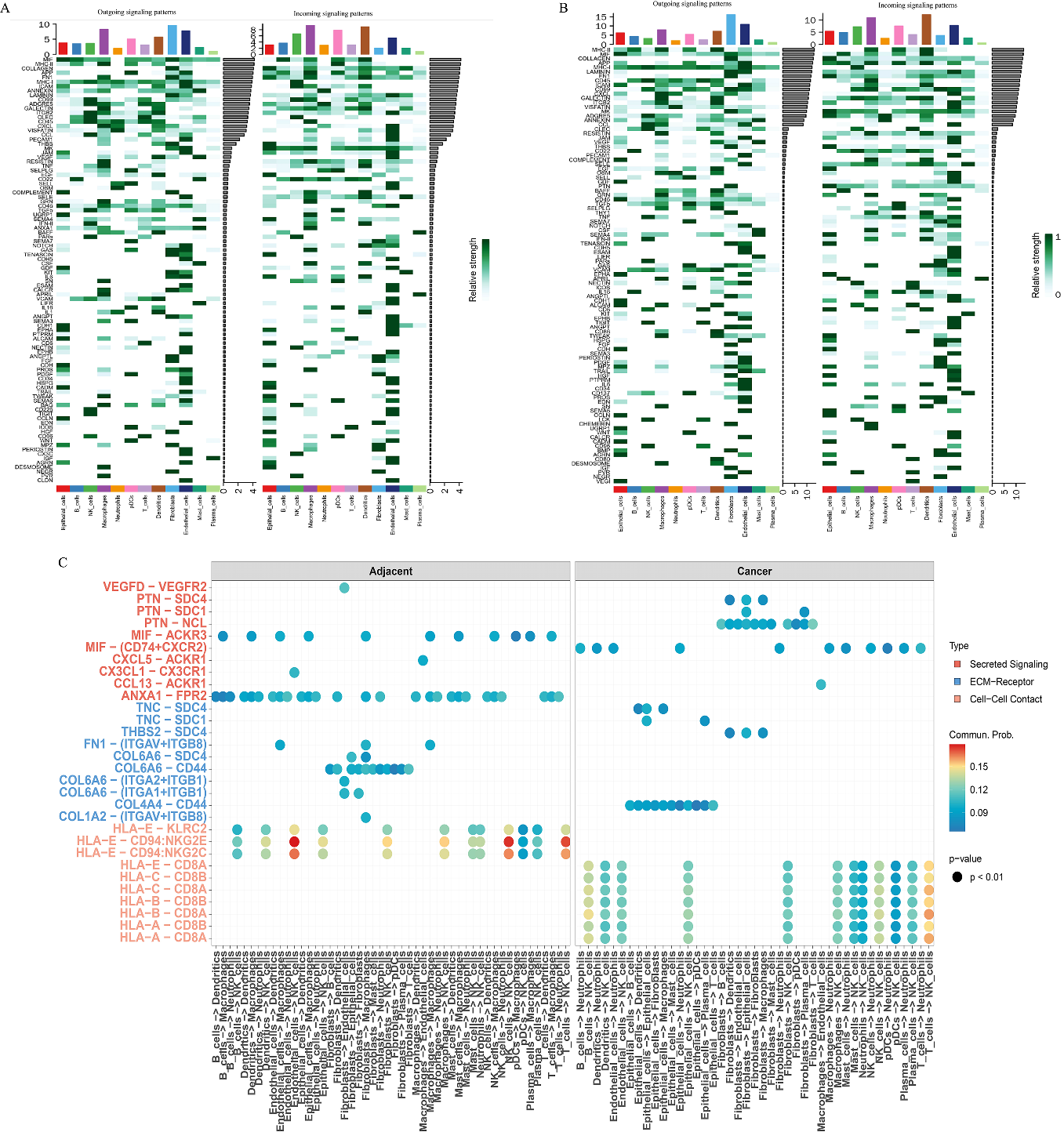


**Supplementary Figure 2.** Rewiring of signaling patterns and representative ligand–receptor interactions in MIA and matched adjacent tissues. (A) Outgoing and incoming signaling patterns across major cell populations in adjacent tissues. (B) Outgoing and incoming signaling patterns across major cell populations in MIA tissues. Color intensity indicates relative signaling strength. (C) Dot plot showing representative ligand–receptor interactions detected in adjacent and MIA tissues. Dot color represents communication probability, and labeled interaction types include secreted signaling, ECM–receptor interactions, and cell–cell contact.


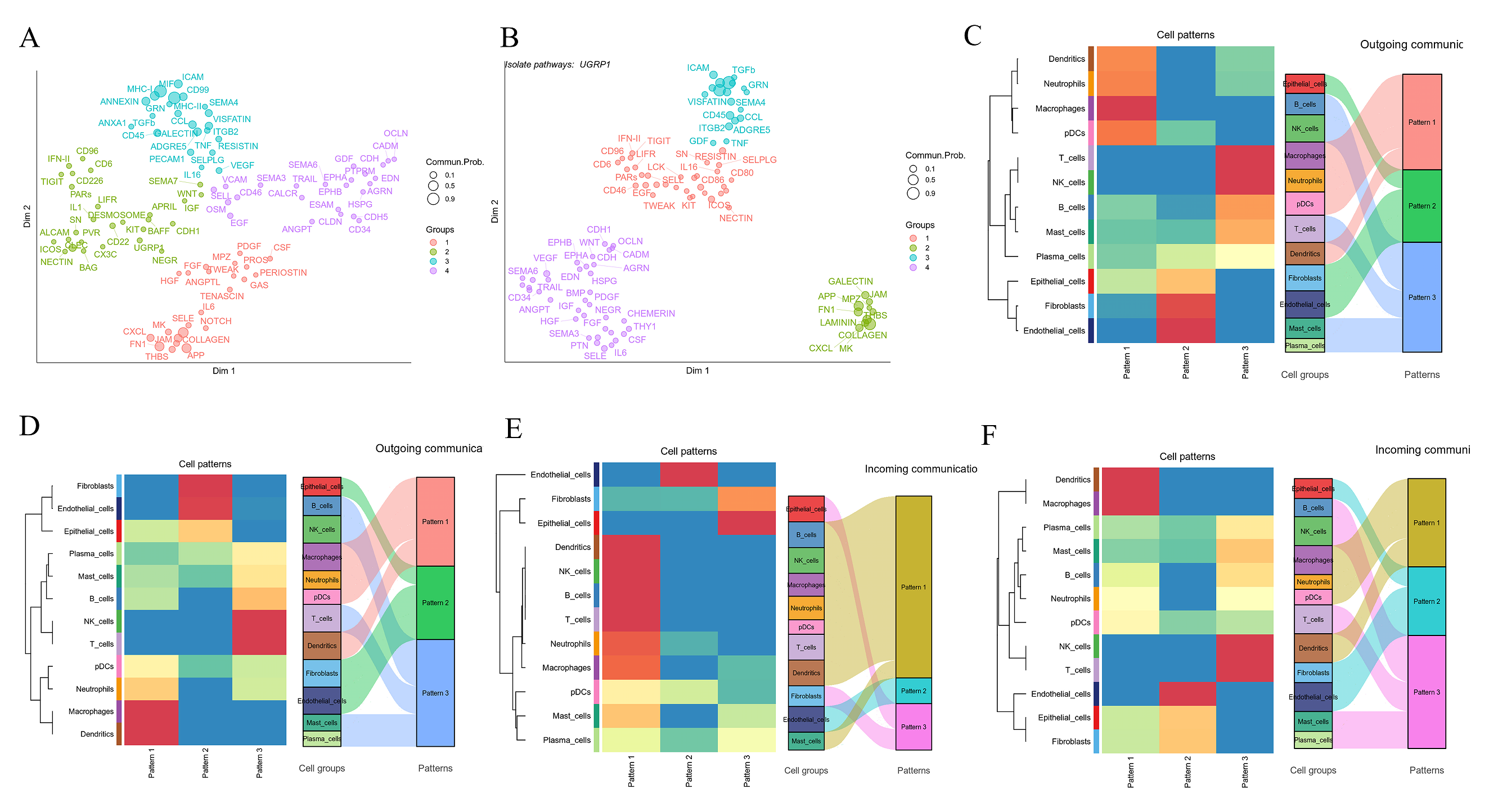


**Supplementary Figure 3.** Outgoing or afferent signals of a population of cells. (A-B) Compared with adjacent tissue, cancer tissue lacks many signaling pathways, but adds additional signaling pathways. (C-D) MIA and adjacent tissues have similar signal output patterns. Cancer and adjacent tissues have different modes of signal reception. (E-F) In adjacent tissues, endothelial cells, fibroblasts and epithelial cells have different signal reception patterns, while these three types of cells in cancer tissues are the same. In adjacent tissues, all immune cells have the same pattern of signal reception, while in cancerous tissues, immune cells are divided into two groups, pattern1 and pattern3.


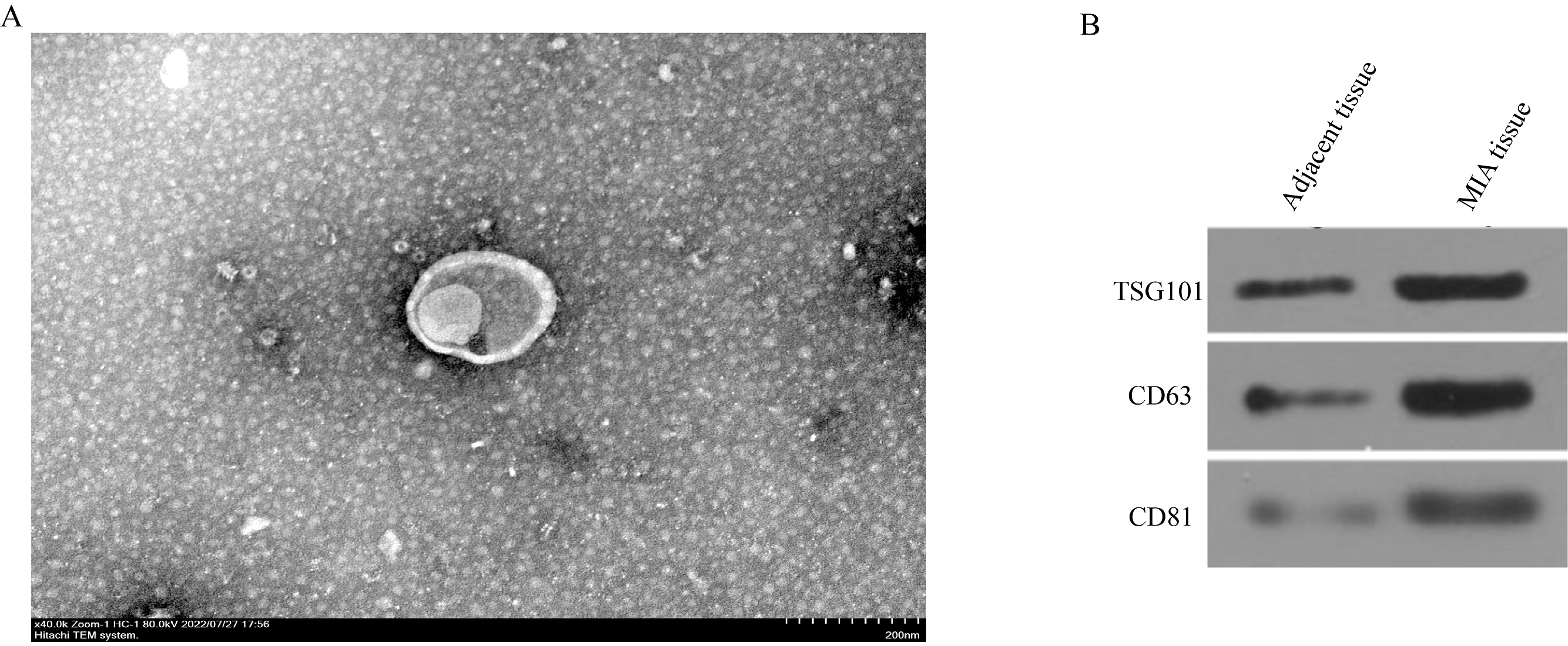


**Supplementary Figure 4.** Validation of exosomes isolated from adjacent and MIA tissues. (A) Representative transmission electron microscopy image of isolated exosomes showing typical vesicular morphology. Scale bar, 200 nm. (B) Western blot detection of the exosomal markers TSG101, CD63, and CD81 in exosome preparations from adjacent tissue and MIA tissue.


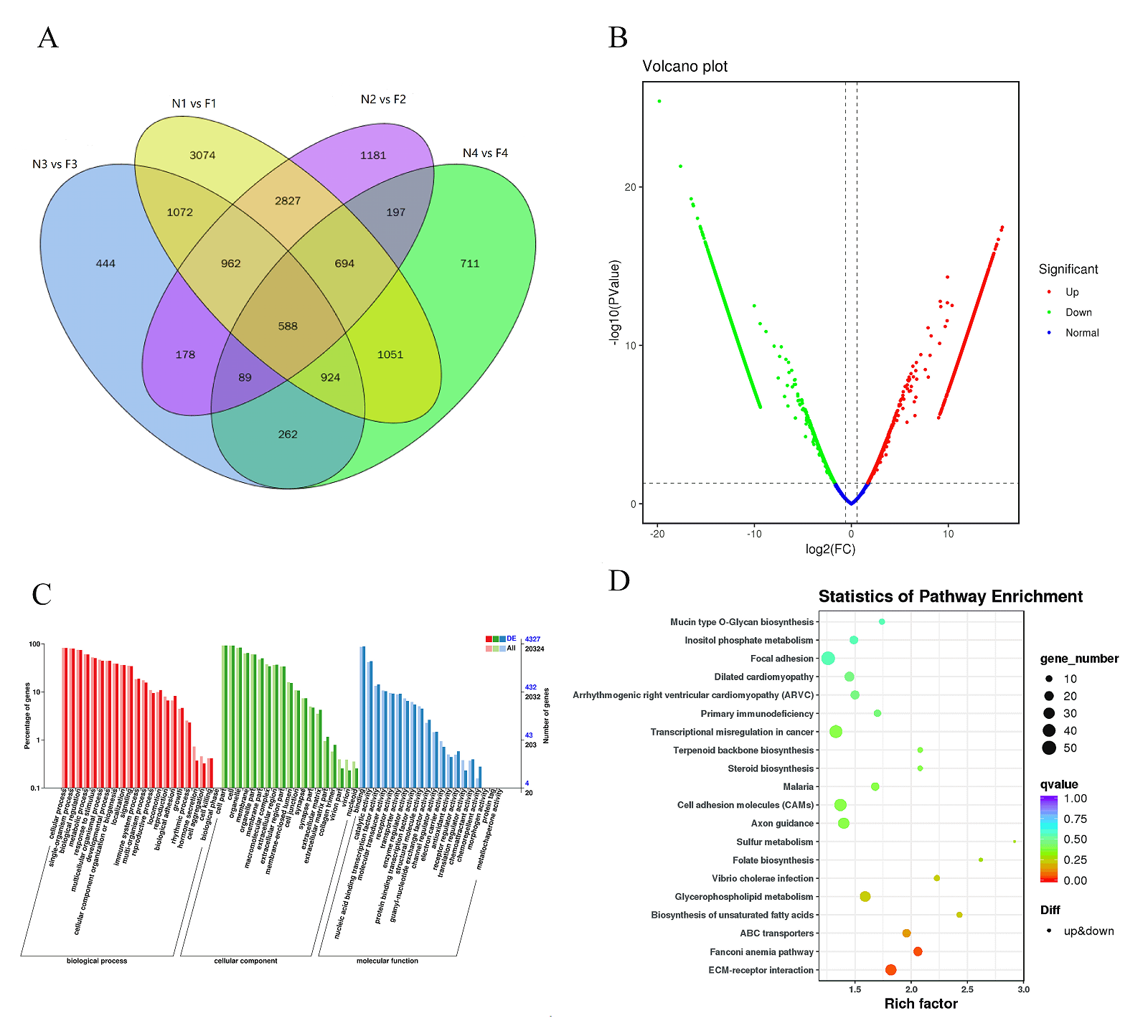


**Supplementary Figure 5.** Results of tissue exosome mRNA differential analysis. (A) Venn diagram of exosomal mRNA differences in each sample. (B) volcano diagram. (C) GO analysis of exosomal mRNA expression in MIA tissues. and (D) Pathway enrichment analysis of exosomal mRNA expression in MIA tissues.


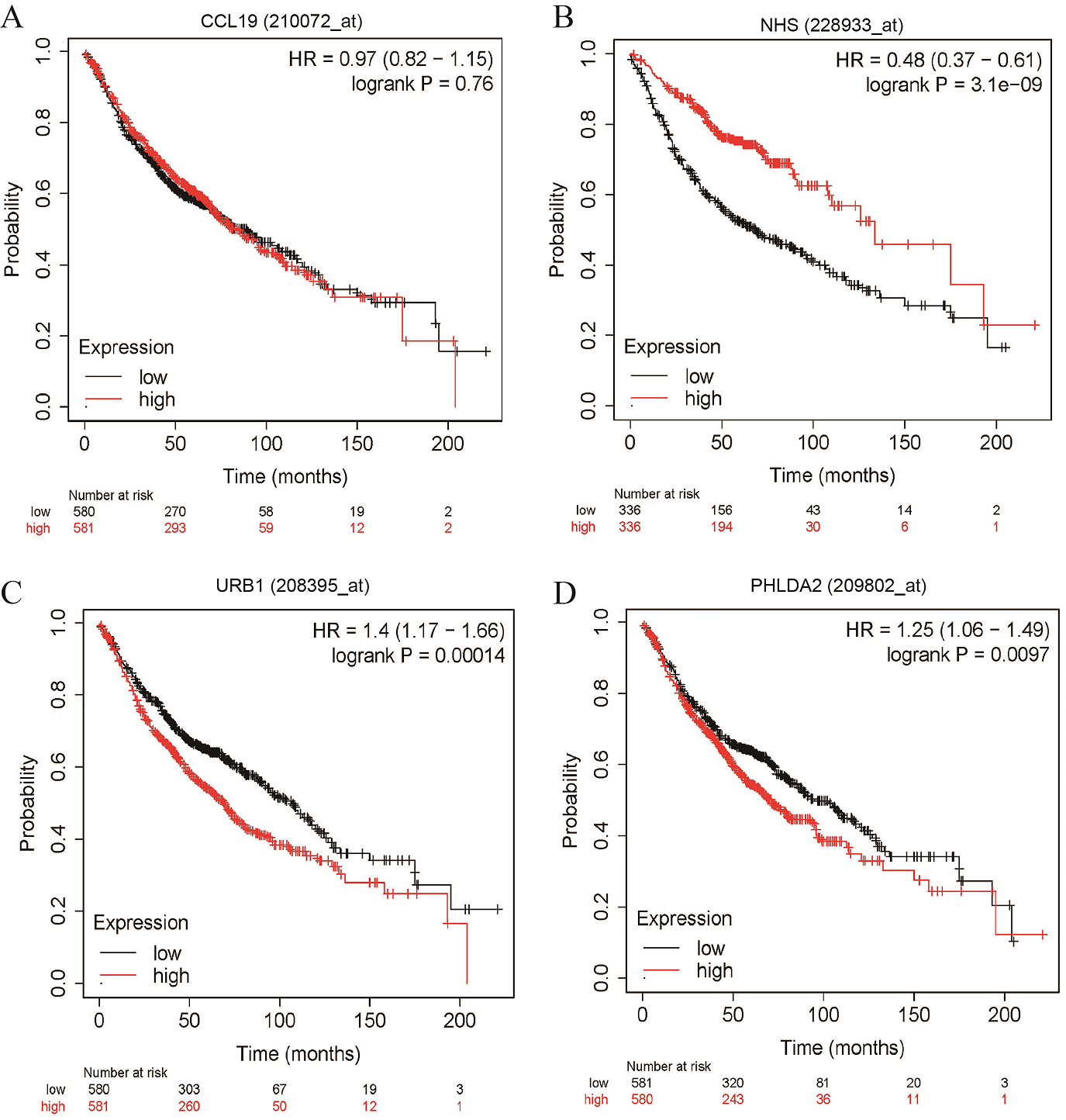


**Supplementary Figure 6.** Prognostic value of exosome-derived candidate genes in LUAD. (A-D) Kaplan–Meier overall survival curves according to the expression of CCL19 (A), NHS (B), URB1 (C), and PHLDA2 (D) in the LUAD cohort.


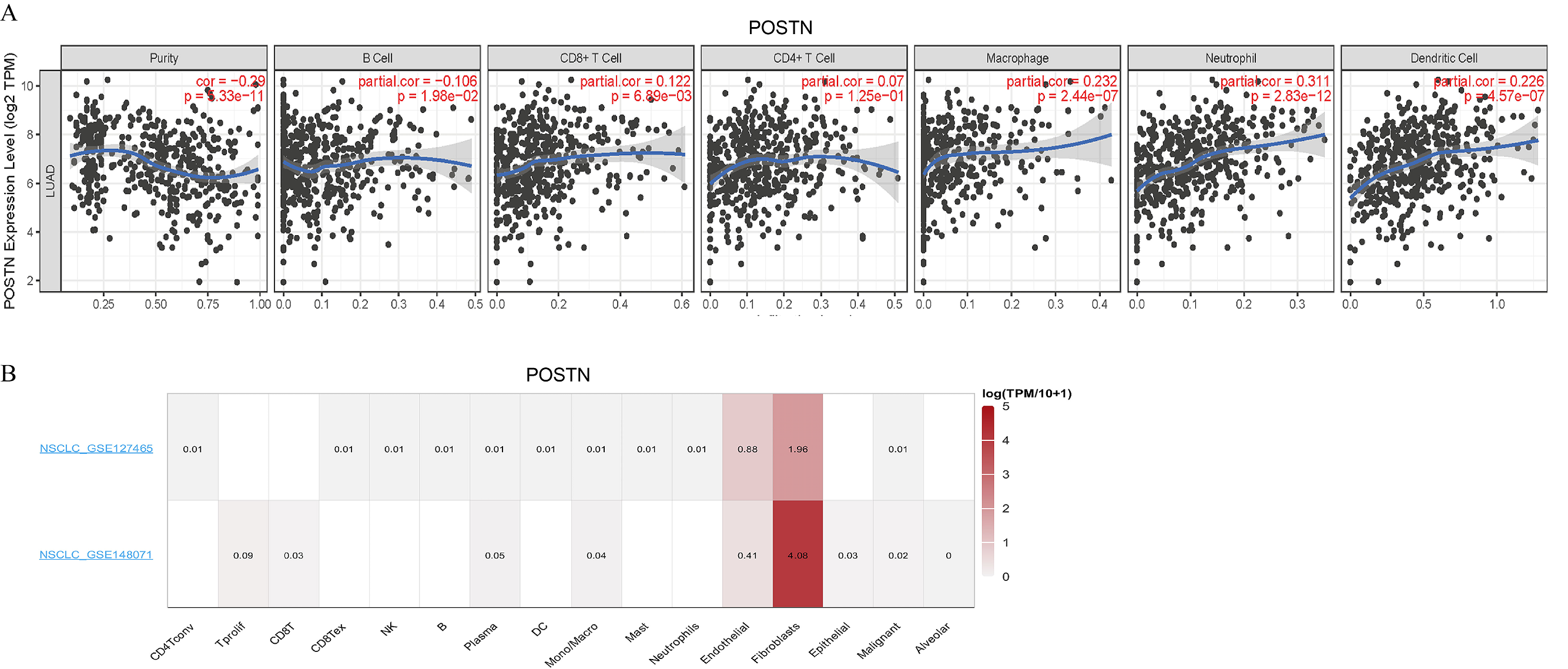


**Supplementary Figure 7.** Evidence supporting fibroblasts as a major source of POSTN in the LUAD microenvironment. (A) Correlation analysis between POSTN expression and tumor purity or immune cell infiltration in LUAD, including B cells, CD8⁺ T cells, CD4⁺ T cells, macrophages, neutrophils, and dendritic cells. (B) Heatmap showing POSTN expression across cell populations in the NSCLC_GSE127465 and NSCLC_GSE148071 single-cell datasets. Color intensity indicates relative expression level.

**Supplementary Table**

**Supplementary Table 1.** Clinical characteristics of the four MIA cases used for scRNA-seq and tissue-derived exosome sequencing.

| Case ID | Age (years) | Sex | Smoking status | Histologic diagnosis | Pathologic stage | Definition of adjacent tissue |
| --- | --- | --- | --- | --- | --- | --- |
| MIA-1 | 61 | Female | Never-smoker | Microinvasive adenocarcinoma | Stage IA1 | Macroscopically normal lung tissue collected ≥2 cm from the tumor margin and histologically confirmed to be free of tumor involvement |
| MIA-2 | 74 | Female | Never-smoker | Microinvasive adenocarcinoma | Stage IA1 | Macroscopically normal lung tissue collected ≥2 cm from the tumor margin and histologically confirmed to be free of tumor involvement |
| MIA-3 | 53 | Female | Never-smoker | Microinvasive adenocarcinoma | Stage IA1 | Macroscopically normal lung tissue collected ≥2 cm from the tumor margin and histologically confirmed to be free of tumor involvement |
| MIA-4 | 68 | Female | Never-smoker | Microinvasive adenocarcinoma | Stage IA1 | Macroscopically normal lung tissue collected ≥2 cm from the tumor margin and histologically confirmed to be free of tumor involvement |

Pathologic staging was assigned according to the AJCC 8th edition. All four cases were used for both scRNA-seq and tissue-derived exosome sequencing.
